# Establishment of a novel gene panel for prognosis assessment in patients with lower grade glioma

**DOI:** 10.1097/MD.0000000000047829

**Published:** 2026-02-28

**Authors:** Bin Zhou, Jianxiong He, Yu Yang, Wei Luo, Bin Xi

**Affiliations:** a Department of Neurosurgery, Jiangxi Provincial People’s Hospital, The First Affiliated Hospital of Nanchang Medical College, Nanchang, Jiangxi, China; b Xiangya Hospital, Central South University, Jiangxi (National Regional Center for Neurological Diseases), Nanchang, Jiangxi, China; c Department of Neurosurgery, Jiangxi Chest Hospital, Nanchang, Jiangxi, People’s Republic of China.

**Keywords:** gene signature, lower grade glioma, nomogram, overall survival, relapse-free survival

## Abstract

Lower-grade gliomas (LGGs) encompass a diverse group of primary brain tumors and are associated with poor survival outcomes, particularly among young adults. This study aimed to develop a novel approach for accurately predicting prognosis in LGG patients. Methods: Using gene expression and clinical data from The Cancer Genome Atlas and the Chinese Glioma Genome Atlas, we identified and validated genes with prognostic significance. We then constructed a 71-gene prognostic score and developed a corresponding nomogram. These tools were evaluated for their association with overall survival (OS) and relapse-free survival (RFS) in LGG patients. Additionally, hierarchical clustering of the 71 genes was performed to identify distinct patient subgroups with unique clinical characteristics. Results: The 71-gene score was found to be a significant and independent predictor of poor OS and RFS in LGG patients, regardless of clinicopathological features. Hierarchical clustering revealed 3 distinct patient subgroups. Notably, tumors in Cluster 2 were characterized by higher tumor grade, more frequent radiation therapy, and worse OS and RFS compared to those in Clusters 1 and 3. Furthermore, the 71-gene nomogram, which incorporates survival-related clinical variables, showed high predictive accuracy for OS and for 3- and 5-year survival rates, with area under the curve values of 0.83, 0.88, and 0.86, respectively. Conclusions: The 71-gene nomogram shows significant potential to enhance prognostic prediction in LGG, offering a valuable and reliable tool for clinicians and researchers.

## 1. Introduction

Gliomas are malignant tumors that arise from glial cells and constitute ~30% of all primary brain and central-nervous-system neoplasms. In the United States the incidence is approximately 6.03 per 100,000 individuals,^[1]^ whereas in China the rate is estimated at 1 to 4 per 100,000.^[2]^ Gliomas encompass various subtypes, including astrocytomas, brain stem gliomas, ependymomas, mixed gliomas, and oligodendrogliomas, with astrocytoma being the most prevalent histological subtype.^[1]^ Gliomas are classified into 4 grades based on their histopathological features, clinical criteria, and molecular parameters.^[3]^ Lower-grade gliomas (LGG) refer specifically to gliomas of World Health Organization grades II and III.^[1,3]^ Although LGG patients have a better prognosis than those with high-grade lesions, a large number of patients experience malignant progression within 10 years, underscoring the crucial need for accurate prognosis prediction.^[4]^

The treatments for glioma patients include surgical resection, radiotherapy or temozolomide treatment.^[5]^ Despite these interventions, the average overall survival (OS) for LGG patients is approximately 7 years, and only 20% of these patients achieve long-term survival.^[5]^ Several studies have established genetic prognostic markers for LGG patients. For example, patients with 1p-19q co-deletion have been associated with prolonged chemotherapy response and improved survival in oligodendrogliomas.^[6]^ *Isocitrate dehydrogenase 1 (IDH1*) mutations are indicative of a more favorable prognosis and a higher response rate to temozolomide in LGG.^[7]^ Patients with glioblastomas with O(6)-methylguanine-DNA methyltransferase promoter methylation are more likely to benefit from concomitant chemoradiotherapy in glioblastomas.^[8]^ Recent studies have uncovered gene expression profiles that have successfully classified patients into subgroups of glioma patients with distinct survival probabilities.^[9,10]^ Furthermore, recent advances further include multi-omics-driven drug discovery,^[11,12]^ microbiome modulation in the tumor microenvironment (TME)^[13]^ and prognosis prediction.^[14]^

In the present study, we aimed to establish a robust and accurate risk score model to effectively predict OS and relapse-free survival (RFS) in LGG patients. We first identified survival-related genes using the expression and clinical data of LGG patients from The Cancer Genome Atlas (TCGA) database^[15]^ and the Chinese Glioma Genome Atlas (CGGA) database.^[16]^ A 71-gene signature was then derived by least absolute shrinkage and selection operator (LASSO) regression, and the pathways underlying the signature were interrogated. Finally, the prognostic value of the 71-gene score was validated in an external set of 182 LGG samples from the CGGA database.^[16]^

## 2. Methods and materials

### 2.1. Acquisition of gene expression and clinical data

RNA-seq profiles and corresponding clinical annotations for 506 LGG samples were downloaded from TCGA. An independent Chinese cohort of 420 LGG samples was obtained from the CGGA, and a further 182 CGGA samples were reserved for external validation. Because all data are publicly available, ethics approval from Jiangxi Provincial People’s Hospital was not required.

### 2.2. Identification of the survival-related genes

RNA-seq data and clinical information for LGG samples were obtained from the TCGA database, totaling 506 samples. Additionally, a dataset consisting of 420 samples was sourced from the CGGA database. An external validation dataset comprising 182 samples was obtained from the CGGA database. As all data were retrieved from publicly available databases, ethics approval from Jiangxi Provincial People’s Hospital was not required for this study.^[17,18]^ Genes significantly linked to clinical outcome were then entered into a multivariate Cox model that included clinical covariates to verify independent prognostic value. Transcripts with a hazard ratio > 1 were classified as risk genes, whereas those with 0 < hazard ratio ≤ 1 were labeled protective genes. Finally, the Immuno-Oncology Biological Research^[19]^ pipeline was applied to the 182-sample validation set to relate these prognostic genes to the TME. Continuous variables and survival differences among molecular subgroups were evaluated with the Kruskal–Wallis *H* test or log-rank test as appropriate.

### 2.3. The establishment and validation of the 71-gene score

Since the gene expression units differed between TCGA and CGGA cohorts, the gene expression data were initially normalized using the z-score formula: *z* = $(x-\bar{x})/s$, where *x* is the gene expression value, $\bar{x}$ is the mean, and *s* is the standard deviation. Then, we performed 10-fold cross-validation of the LASSO model using the R package (Stanford University, Stanford) glmnet to determine the optimal combination of genes for predicting OS in the TCGA dataset.^[20]^ The 71-gene score = expression of gene 1 × β1 + expression of gene 2 × β2 +⋯+ expression of gene n × βn. The β values are the regression coefficients derived from the LASSO model. We employed the same method as that used to identify survival-related genes to study the correlations between OS, RFS, and the 71-gene score. The correlations between clinical characteristics and the 71-gene score were analyzed using a linear regression model in both the TCGA (n = 506) and CGGA (n = 420) cohorts. Statistical significance was set at *P* < .05.

### 2.4. Gene set enrichment analysis (GSEA)

GSEA^[21]^ was performed to analyze the altered Kyoto Encyclopedia of Genes and Genomes (KEGG) signaling pathways with the default parameters between the high 71-gene score and low 71-gene score groups divided by the median 71-gene score. Statistical significance was set at *P* value < .05.

### 2.5. Unsupervised hierarchical clustering analysis

Unsupervised hierarchical clustering of the 71-gene signature was performed in both the TCGA and CGGA cohorts with the R package pheatmap.^[22]^ Clinical characteristics across the resulting subgroups were compared with Student *t* test for continuous variables and Fisher exact test for categorical variables. Survival curves were generated with the survival package^[17]^ and compared among the 3 subgroups by the log-rank test; *P* < .05 was considered statistically significant.

### 2.6. Construction and validation of the 71-gene nomogram

A nomogram predicting OS was constructed in the TCGA cohort with the rms package in R, integrating patient age, histologic grade, IDH1 mutation status, and the 71-gene risk score – each independently associated with OS. Model performance was quantified by area under the curve (AUC) for OS, RFS, 3-year survival, and 5-year survival, and was externally validated in both the CGGA dataset and the independent 182-sample validation set using the pROC package.

## 3. Results

### 3.1. Analysis of clinical factors

In the TCGA cohort, patient age, histological subtype, grade, tumor weight, IDH1 mutation status and receipt of targeted therapy were all significantly associated with OS (*P* < .05, Student *t* test or Fisher exact test as appropriate; Table 1). Similarly, in the CGGA cohort, histological grade, IDH1 mutation status, MGMT promoter methylation and 1p/19q co-deletion correlated significantly with OS (*P* < .05, Fisher exact test; Table S1, Supplemental Digital Content, https://links.lww.com/MD/R451). With respect to RFS, IDH1 mutation was the only clinicopathological factor predictive of longer RFS in the TCGA cohort (*P* < .05, Fisher exact test; Table 1), whereas histological grade and chemotherapy showed no significant association with RFS in the CGGA dataset (*P* > .05 for both, Fisher exact test or Student *t* test as appropriate; Table S1, Supplemental Digital Content, https://links.lww.com/MD/R451).

**Table 1:** Association between the clinicopathologic characteristics and patients’ OS as well as RFS in the TCGA dataset.

Variables	Group	Alive	Dead	*P* value	Non-relapse	Relapse	*P* value	Statistical method
Age		41.68	49.06	≤.001	42.09	43.62	.29	Student *t* test
Tumour weight		338.38	285.01	.02	331.86	328.05	.89	Student *t* test
Gender	Female	179	45	.24	123	60	.83	Fisher exact test
	Male	237	45		150	69		
History of cancer	No	183	27	.34	122	49	.3	Fisher exact test
	Yes	107	22		76	40		
Histological type	Astrocytoma	151	41	.17	94	54	.26	Fisher exact test
	Oligoastrocytoma Oligodendroglioma	112 153	17 32		77 102	28 47		
Histologic grade	G2	217	26	≤.001	140	57	.20	Fisher exact test
	G3	198	64		132	72		
*IDH1* mutation	Wild-type	79	34	≤.001	44	41	≤.001	Fisher exact test
	Mutant	337	56		229	88		
*TP53* mutation	Wild-type Mutant	211 205	47 43	.82	139 134	57 72	.24	Fisher exact test
Radiation therapy	No Yes	104 116	14 24	.29	65 81	28 33	.88	Fisher exact test
Targeted therapy	No Yes	167 199	22 48	.03	124 145	46 80	.08	Fisher exact test

OS = overall survival, RFS = relapse-free survival, TCGA = The Cancer Genome Atlas.

### 3.2. Analysis of prognosis-related genes

**Figure 1.**
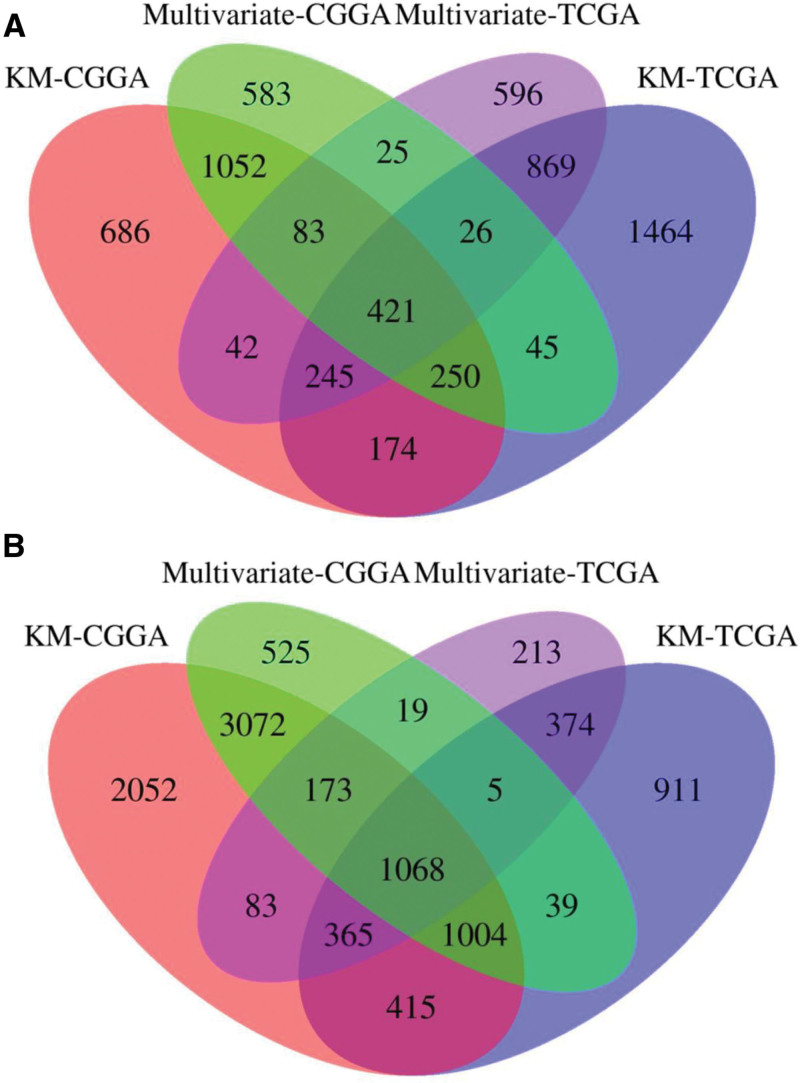
Identification of common survival-related genes in the TCGA and CGGA datasets. (A) Protective genes shared by TCGA and CGGA cohorts: this panel depicts the protective genes that were commonly identified in both the TCGA and CGGA datasets as significantly associated with OS. The genes marked in red indicate those that were significantly related to OS in the CGGA cohort using Kaplan–Meier analysis, while the genes in blue represent those identified in the TCGA cohort. (B) Risk genes shared by TCGA and CGGA cohorts: this panel shows the risk genes that were commonly found in both the TCGA and CGGA datasets to be significantly related to OS. The genes highlighted in green represent those that were confirmed to be significantly associated with OS in the CGGA cohort through multivariate analysis, while the purple genes were confirmed in the TCGA cohort. CGGA = Chinese Glioma Genome Atlas, OS = overall survival, TCGA = The Cancer Genome Atlas.

Kaplan–Meier analysis of the TCGA discovery cohort identified 3 494 genes whose high expression was associated with favorable OS and 4 181 genes linked to poor OS (*P* < .05, log-rank test; Fig. 1). Multivariate Cox regression that included clinical covariates retained 1561 protective and 1812 risk genes (*P* < .05; Table S2, Supplemental Digital Content, https://links.lww.com/MD/R451; Fig. 1). We next tested these candidates in the 420-sample CGGA validation set: high expression of 421 genes correlated with prolonged OS, whereas high expression of 1 068 genes predicted shorter OS (*P* < .05; Fig. 1, Table S3, Supplemental Digital Content, https://links.lww.com/MD/R451). To relate these prognostic genes to the TME, we first quantified immune infiltration. Regulatory T cells, CD8 T cells and M2 macrophages were the 3 most abundant populations (Fig. S1, Supplemental Digital Content, https://links.lww.com/MD/R451). Signature scores for several metabolic pathways – including nicotinate/nicotinamide metabolism, glutathione metabolism and terpenoid backbone biosynthesis – were significantly associated with both OS and RFS (Figs. S2 and S3, Supplemental Digital Content, https://links.lww.com/MD/R451). Unsupervised clustering of cellular infiltration patterns yielded 3 TME subgroups. Cluster-1 samples displayed the highest abundance of monocytes and activated memory CD4^+^ T cells, elevated expression of cluster of differentiation 68 and Rho GDP dissociation inhibitor beta, lower scores for the CD8_T_cells_Bindea_et_al and Cytokines_Li_et_al signatures, and significantly worse OS and RFS than cluster-2 or cluster-3 samples (*P* < .05 for all comparisons, Kruskal–Wallis or log-rank test; Figs. S4–S8, Supplemental Digital Content, https://links.lww.com/MD/R451).

### 3.3. The 71-gene score as an indicator of favorable OS

**Figure 2.**
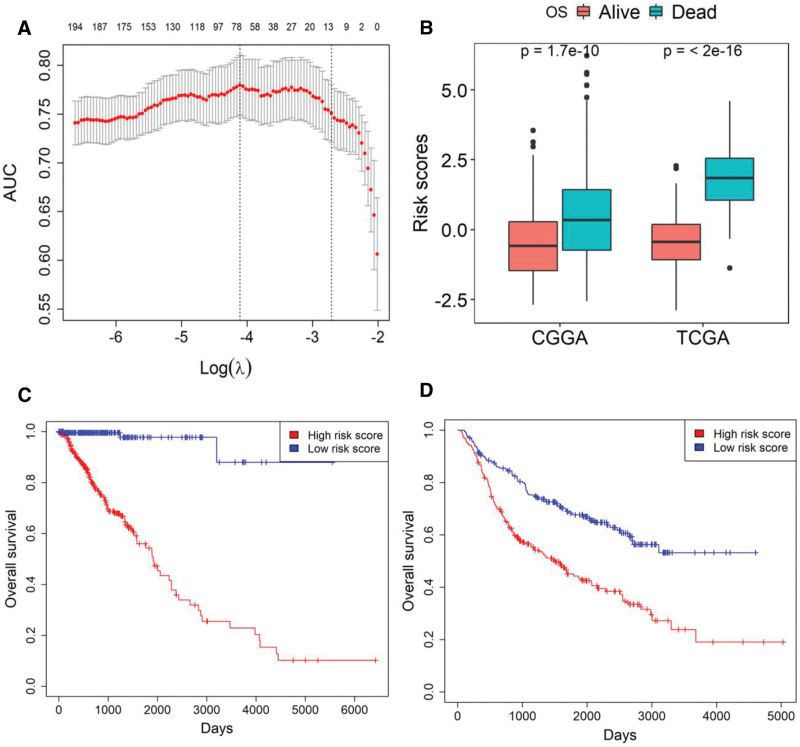
The 71-gene score as a negative prognosticator for overall survival in LGG. (A) Selection of the optimal LASSO model for the 71-gene signature: this plot depicts the relationship between the log-scaled lambda values, the AUC, and the number of genes with coefficients above zero in the LASSO model. The numbers at the top indicate the number of genes with coefficients above zero in the LASSO model. The left and right vertical dotted lines represent the lambda.min and lambda.1se values for λ, respectively. Lambda.min is the value that minimizes the out-of-sample loss in cross-validation, while lambda.1se is the largest lambda value within 1 standard error of the minimum. (B) Differential 71-gene scores between living and deceased LGG patients: this graph compares the 71-gene scores between living and deceased LGG patients in both cohorts, revealing a significant difference. (C) Kaplan–Meier survival analysis in the TCGA cohort: the Kaplan–Meier survival curve demonstrates a significant difference in overall survival between the high and low 71-gene score groups in the TCGA cohort. (D) Kaplan–Meier survival analysis in the CGGA cohort: similarly, the Kaplan–Meier survival curve in the CGGA cohort also shows a significant difference in overall survival between the high and low 71-gene score groups. AUC = area under the curve, CGGA = Chinese Glioma Genome Atlas, LGG = lower grade glioma, OS = overall survival, TCGA = The Cancer Genome Atlas.

Ten-fold cross-validation of the LASSO model identified a 71-gene signature that maximize the AUC at log(λ) = –4.2, yielding optimal prognostic performance (Fig. 2A; Tables S2–S4, Supplemental Digital Content, https://links.lww.com/MD/R451). In both TCGA and CGGA cohorts, deceased patients had significantly higher 71-gene scores than living patients (*P* < .05, Student *t* test; Fig. 2B). Kaplan–Meier analysis showed that high scores were associated with reduced survival probability in TCGA (*P* < .05, log-rank test; Fig. 2C), and multivariate Cox regression confirmed the 71-gene score as an independent predictor of poor OS after adjustment for clinical covariates (*P* < .05; Table 2, Fig. 2D). The inverse relationship was independently validated in the CGGA dataset (*P* < .05; Table 2, Fig. 2D).

**Table 2:** Multivariate analyses between OS and the 71-gene score in the TCGA and CGGA datasets.

TCGA dataset				CGGA dataset			
Clinical feature	HR	95% CI	*P* value	Clinical feature	HR	95% CI	*P* value
Age	1.05	1.03–1.08	<.001	Histologic grade	3.04	2.12–4.37	<.001
Histologic grade	2.25	1.16–4.35	.02	IDH1	0.6	0.43–0.85	<.01
Tumor weight	1	0.99–1	.24	1p-19q codeletion	0.45	0.3–0.67	<.001
IDH1	0.5	0.29–0.88	.02	The 71-gene score	1.88	1.35–2.63	<.001
Targeted therapy	0.66	0.35–1.24	.2				
The 71-gene score	24.32	5.88–100.68	<.001				

CGGA = Chinese Glioma Genome Atlas, CI = confidence interval, HR = hazard ratio, OS = overall survival, RFS = relapse-free survival, TCGA = The Cancer Genome Atlas.

### 3.4. The 71-gene score as a negative predictor for RFS

Because long-term RFS is a critical surrogate for OS, we examined the signature’s relationship with recurrence. Recurrent tumors displayed higher 71-gene scores than nonrecurrent tumors in both TCGA and CGGA (*P* < .05, Student *t* test; Fig. S9A, Supplemental Digital Content, https://links.lww.com/MD/R451). Kaplan–Meier analysis revealed that elevated scores predicted shorter RFS (*P* < .05, log-rank test; Fig. S9B, Supplemental Digital Content, https://links.lww.com/MD/R451), and multivariate Cox modeling showed this association remained significant after adjusting for IDH1 mutation status (*P* < .05; Table S5, Supplemental Digital Content, https://links.lww.com/MD/R451). The adverse correlation was corroborated in the CGGA validation set by both Kaplan–Meier and multivariate analyses (*P* < .05; Fig. S9C, Table S5, Supplemental Digital Content, https://links.lww.com/MD/R451).

### 3.5. The 71-gene score is a robust tool for risk stratification independent of clinical factors

**Figure 3.**
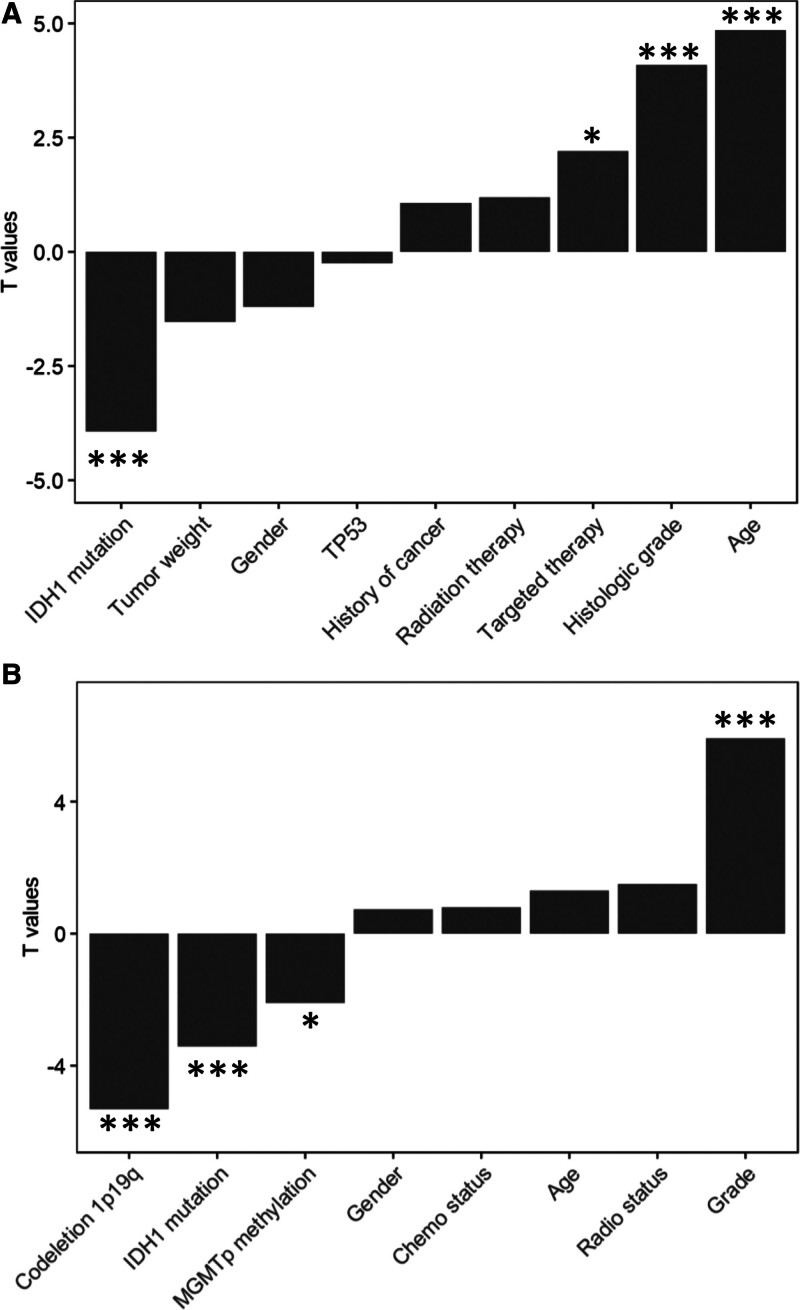
Correlations between the 71-gene score and clinical features. This figure illustrates the relationships between the 71-gene score and various clinical features using linear regression model analysis. Panel (A) shows the correlations in the TCGA cohort, while Panel (B) displays the correlations in the CGGA cohort. Statistical significance is indicated by asterisks, where * represents a *P*-value < .05 and *** denotes a *P*-value < .001. CGGA = Chinese Glioma Genome Atlas, TCGA = The Cancer Genome Atlas.

To further characterize the relationship between the 71-gene score and clinicopathological variables, we fitted linear regression models. In the TCGA cohort, the signature was negatively correlated with IDH1 mutation and positively correlated with patient age and histological grade (*P* < .05; Fig. 3A); these associations were recapitulated in the CGGA cohort (*P* < .05; Fig. 3B). We next investigated whether the 71-gene score retains prognostic value within individual clinical strata. Kaplan–Meier analysis showed that a high score predicted inferior OS irrespective of sex, age, tumor weight, histological grade, TP53 or IDH1 mutation status, prior malignancy, radiotherapy or targeted therapy in both TCGA (*P* < .05, log-rank test; Figs. S10–S12, Supplemental Digital Content, https://links.lww.com/MD/R451) and CGGA (*P* < .05, log-rank test; Figs. S13–S15, Supplemental Digital Content, https://links.lww.com/MD/R451) datasets. Similarly, a high 71-gene score was associated with shorter RFS across all clinical subgroups examined (*P* < .05, log-rank test; Table S6, Supplemental Digital Content, https://links.lww.com/MD/R451).

### 3.6. Identification of signaling pathways associated with the 71-gene score

**Figure 4.**
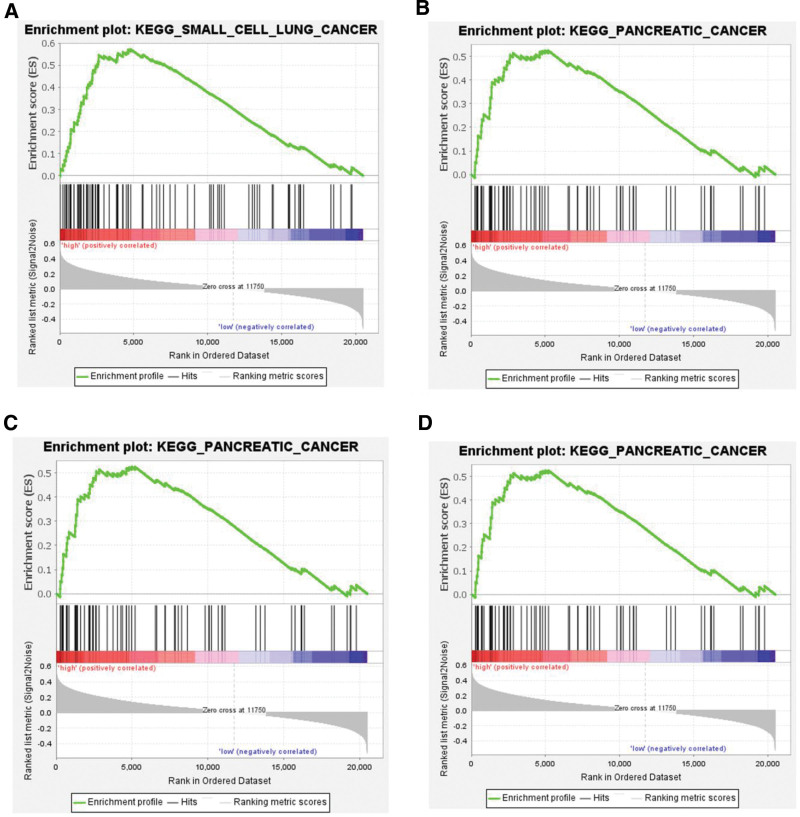
KEGG pathways contributing to the prognostic value of the 71-gene score. Utilizing gene set enrichment analysis based on the TCGA dataset, this figure highlights several key KEGG pathways that are significantly associated with the 71-gene score. Panel (A–D) reveal the small cell lung cancer, pancreatic cancer, mismatch repair, focal adhesion signaling pathways, which are enriched in the high 71-gene score group (n = 253). KEGG = Kyoto Encyclopedia of Genes and Genomes, TCGA = The Cancer Genome Atlas.

Samples were split at the median 71-gene score and GSEA was run between high- and low-risk tumors. In TCGA, small-cell lung cancer, pancreatic cancer, mismatch-repair and focal-adhesion pathways were significantly up-regulated in the high-score group (*P* < .05; Fig. 4). In the CGGA cohort, 32 KEGG pathways were enriched in high-score tumors; the top 5 were “pathways in cancer,” ubiquitin-mediated proteolysis, the Wingless and INT1 (Wnt) signaling, small-cell lung cancer and tight junction (*P* < .05 for each; Table S7, Supplemental Digital Content, https://links.lww.com/MD/R451). These data suggest that the 71-gene signature tracks survival because it captures activity of key oncogenic circuits.

### 3.7. Patient stratification with the 71-gene panel

**Figure 5.**
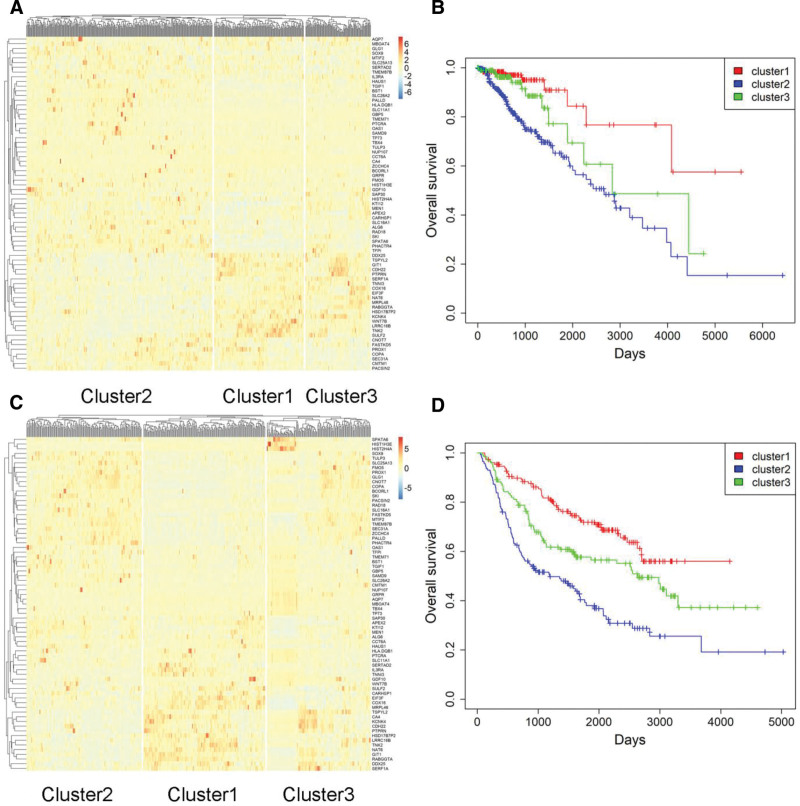
Unsupervised hierarchical clustering of the 71-gene panel reveals 3 distinct classes of LGG patients. (A) Hierarchical clustering of the 71-gene panel in the TCGA dataset identified 3 distinct classes of LGG patients. (B) Kaplan–Meier survival analysis revealed significant differences in overall survival among the 3 classes of LGG patients identified in the TCGA cohort. (C) Similarly, unsupervised hierarchical clustering of the 71-gene panel in the CGGA dataset also uncovered 3 distinct classes of LGG patients. (D) The overall survival of the 3 classes of LGG patients in the CGGA cohort also exhibited significant differences, as demonstrated by the Kaplan–Meier survival analysis. CGGA = Chinese Glioma Genome Atlas, LGG = lower grade glioma, TCGA = The Cancer Genome Atlas.

Unsupervised hierarchical clustering of the 71-gene signature in TCGA revealed 3 robust patient subsets (Fig. 5A). Cluster-2 tumors were of higher grade, were irradiated more frequently, and were associated with shorter OS and RFS than cluster-1 or cluster-3 tumors (*P* < .05, Fisher exact or log-rank test; Fig. 5B, Fig. S16A, Table S8, Supplemental Digital Content, https://links.lww.com/MD/R451). An equivalent 3-class structure was recovered in CGGA (Fig. 5C). Again, cluster-2 displayed the highest 71-gene scores, the lowest frequency of 1p/19q co-deletion, the highest grade relative to cluster-3, and the poorest OS and RFS (*P* < .05 for all comparisons, Student’s *t*, Fisher exact or log-rank test; Fig. 5D, Fig. S16B, Table S9, Supplemental Digital Content, https://links.lww.com/MD/R451). Thus, the 71-gene signature reproducibly segregates LGG patients into biologically and clinically distinct prognostic groups.

### 3.8. Nomogram combining 71-gene signature and clinical-related variables predicts patients’ prognosis

**Figure 6.**
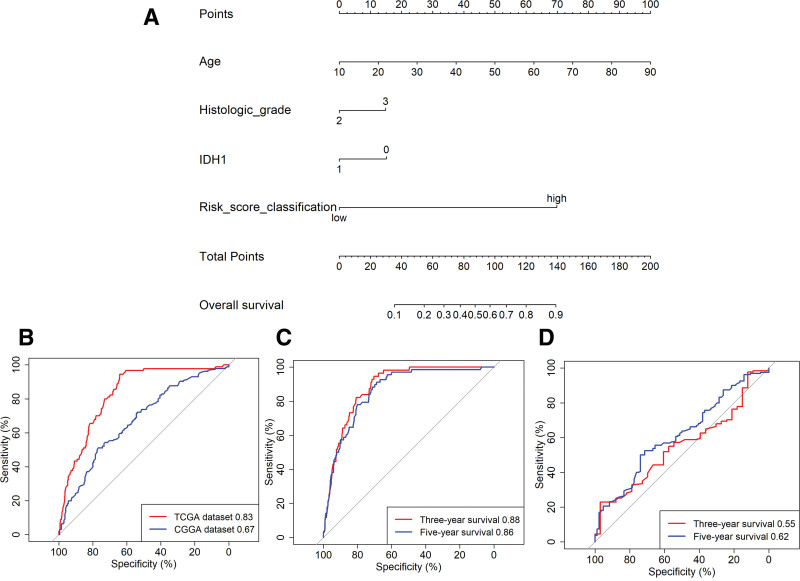
The 71-gene nomogram for predicting disease risk in LGG patients. (A) This mRNA-based nomogram predicts OS in LGG patients. In this nomogram, histologic grades 2 and 3 refer to grade II and III gliomas, respectively. The 71-gene score is stratified into high and low categories based on the median 71-gene score, with “high” and “low” representing scores above and below the median, respectively. (B) The ROC plot demonstrates the predictive accuracy of the nomogram for overall survival in both the TCGA and CGGA cohorts. (C) The ROC plot illustrates the predictive performance of the nomogram for 3-year and 5-year overall survival specifically in the TCGA cohort. (D) Similarly, the ROC plot shows the predictive ability of the nomogram for 3-year and 5-year overall survival in the CGGA cohort. CGGA = Chinese Glioma Genome Atlas, LGG = lower grade glioma, OS = overall survival, ROC = receiver operator characteristic, TCGA = The Cancer Genome Atlas.

In both TCGA and CGGA cohorts, patient age, histologic grade, IDH1 mutation status, and the 71-gene signature were independently associated with OS. We therefore integrated these variables into a 71-gene nomogram (Fig. 6A). Receiver-operating-characteristic analysis showed that the model accurately predicted OS and 3- and 5-year survival rates in TCGA (AUC 0.83, 0.88, and 0.86, respectively; Fig. 6B, C). The discriminative capacity was confirmed in CGGA, although AUCs were lower (0.67, 0.55, and 0.62; Fig. 6B, D), underscoring the clinical utility of the nomogram for estimating OS in LGG. A parallel 71-gene nomogram for RFS yielded AUCs of 0.59, 0.55, and 0.53 for RFS and 3- and 5-year RFS, respectively (Fig. S17, Supplemental Digital Content, https://links.lww.com/MD/R451).

### 3.9. External validation of the prognostic value of the 71-gene signature in LGG

**Figure 7.**
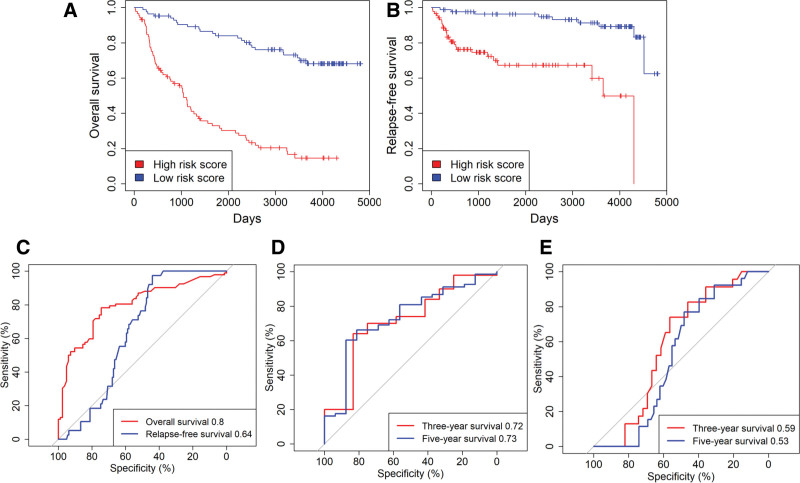
External validation of the prognostic value of 71-gene signature in LGG. (A–B) The Kaplan–Meier survival curves demonstrate significant negative correlations between OS(A), RFS(B) and the 71-gene scores in the external validation dataset. (C) The ROC plot demonstrates the predictive accuracy of the nomogram for OS and RFS in the external validation dataset. (D) The ROC plot illustrates the predictive performance of the nomogram for 3- and 5-year OS specifically in the external validation dataset. (E) Similarly, the ROC plot shows the predictive ability of the nomogram for 3-year and 5-year RFS in the external validation dataset. LGG = lower grade glioma, OS = overall survival, ROC = receiver operator characteristic.

To confirm the prognostic value of the 71-gene signature in LGG, we analyzed an external validation set of 182 samples from the CGGA database and computed 71-gene scores with the LASSO-derived coefficients. Kaplan–Meier and multivariate Cox analyses again showed that higher scores were significantly associated with shorter overall and RFS (Fig. 7A, B and Table 3). The 71-gene nomogram retained good discrimination in this independent cohort, with AUCs of 0.80, 0.72, and 0.73 for OS and 3- and 5-year survival, respectively (Fig. 7C, D). Discrimination for RFS was also validated, yielding AUCs of 0.64, 0.59 and 0.53 for RFS and 3- and 5-year RFS, respectively (Fig. 7C, E).

**Table 3:** Multivariate analyses between OS, RFS and the 71-gene score in the external CGGA dataset.

OS				RFS			
Clinical feature	HR	95% CI	*P* value	Clinical feature	HR	95% CI	*P* value
Histologic grade	2.79	1.75–4.46	<.001	Histologic grade	5.46	2.23–13.39	<.001
IDH1	0.84	0.53–1.34	.46	Chemotherapy	0.69	0.31–1.52	.35
1p-19q codeletion	0.24	0.12–0.5	<.001	The 71-gene score	4.92	2–12.11	<.001
The 71-gene score	2.68	0.31–0.41	<.001				

CGGA = Chinese Glioma Genome Atlas, CI = confidence interval, HR = hazard ratio, OS = overall survival, RFS = relapse-free survival.

## 4. Discussion

Despite noteworthy advancements in the treatment of LGG over the past decade, the global mortality rate associated with these tumors remains comparatively high. Numerous studies have established a link between molecular tumor markers and gene signatures and OS in LGG patients.^[6,23–26]^ In the present study, we developed a 71-gene score model utilizing a linear combination of 71 gene expression levels weighted by the β-values derived from the LASSO model. After accounting for established clinical factors, we found a negative correlation between the 71-gene score and OS as well as RFS. Compared to traditional prognosticators such as histologic grade, 1p-19q co-deletion, and *IDH1* mutation status, our 71-gene expression signature offers distinct advantages. Notably, our model accurately predicted OS and RFS, irrespective of clinicopathological characteristics. In addition, we constructed a 71-gene nomogram that incorporated survival-related clinical factors and a 71-gene signature. This 71-gene nomogram may serve as an improved risk classification tool for patients with wild-type 1p-19q co-deletion and *IDH1* mutation status, or those with the same histological grade.

Furthermore, we evaluated the clinical significance of the 71-gene score and identified relevant KEGG pathways. Notably, the small cell lung cancer, pancreatic cancer, mismatch repair, focal adhesion signaling pathways were significantly upregulated in the high 71-gene score group of TCGA cohort. Moreover, in the high 71-gene group of CGGA cohort, genes involved in the top 5 significantly upregulated pathways were pathways in cancer, ubiquitin mediated proteolysis, Wnt signaling pathway, small cell lung cancer, and tight junction. The Wnt signaling pathway plays a pivotal role in the development of the central nervous system. Aberrant activation of this pathway has been linked to malignant transformation and development of brain tumors in the nervous system.^[27,28]^ Based on our findings, we hypothesized that the Wnt signaling pathway and the aforementioned signaling pathways are integral components of the molecular mechanism underlying the 71-gene score and survival outcomes in LGG patients.

The 71 genes we identified exhibited diverse functional roles in cancer tumorigenesis. For instance, cadherin-like protein 22 (*CDH22*) is a transmembrane glycoprotein that plays a critical role in cell-cell adhesion and metastasis and is associated with morphogenesis and tissue formation in the brain and neuroendocrine organs.^[29–31]^ Notably, CDH22 protein levels were downregulated in the breast cancer tissues and melanoma samples. Consistent with our findings, dysregulated *CDH22* expression has been identified as an independent predictor of poor survival in breast cancer^[32]^ and melanoma.^[29]^ The eukaryotic initiation factor 3f (eIF3f) is the p47 subunit of the multi-subunit eIF3 complex, which is essential for translation initiation.^[33]^ *EIF3f* expression is significantly downregulated in many human cancers.^[34,35]^ Notably, increased *eIF3f* expression is associated with a favorable prognosis in gastric cancer.^[34]^ Its enhanced expression significantly represses cancer cell growth and tumorigenic ability in nude mice.^[36]^ Aquaporin 7 (*AQP7*) is a water/glycerol transporter that regulates adipocyte glycerol efflux and affects lipid and glucose homeostasis. Low *AQP7* expression correlates with tumor grade and aggressive features in hepatocellular carcinoma.^[37]^ Furthermore, *AQP7* serves as a prognostic indicator of OS in patients with breast cancer. In mouse breast cancer models, reduced *AQP7* expression leads to decreased primary tumor burden and lung metastasis.^[38]^ These genes not only provide insights into the molecular mechanisms underlying LGG but also present potential targets for developing targeted therapies. For instance, silencing the expression of *eIF3f* or *AQP7* has been shown to significantly reduce cell proliferation and metastasis in various cancer types.^[36,38]^

## 5. Conclusion

In summary, we have identified a 71-gene prognostic signature and constructed a nomogram that significantly improves outcome prediction for patients with LGG. Nevertheless, the study has important limitations. The retrospective use of TCGA and CGGA data exposes the findings to selection, information and treatment-allocation biases, and molecular measurements were performed on different platforms with variable sample processing. Prospective, multi-institutional validation is still lacking, and real-world performance remains untested.

We therefore plan a multicenter observational study that will enroll 500 newly diagnosed LGG patients to evaluate the signature’s ability to predict 3-year progression-free and OS. In parallel, we will develop a targeted RNA-seq assay compatible with FFPE and intraoperative smear specimens, delivering results within 48 hours. Integration of the 71-gene score into routine care will allow escalation of adjuvant therapy for high-score patients and de-escalation for low-score patients, establishing the first molecularly driven, risk-adapted management algorithm for LGG.

## Author contributions

**Conceptualization:** Jianxiong He, Bin Xi.

**Data curation:** Bin Zhou, Jianxiong He, Wei Luo.

**Formal analysis:** Wei Luo.

**Investigation:** Bin Zhou, Yu Yang.

**Methodology:** Bin Zhou, Yu Yang.

**Validation:** Jianxiong He.

**Writing – original draft:** Bin Xi.

**Writing – review & editing:** Jianxiong He, Wei Luo, Bin Xi.

## Supplementary Material


