# Supplementary material for: Establishment of a novel gene panel for prognosis assessment in patients with lower grade glioma
Source: Medicine (Baltimore). 2026 Feb 28;105(9):e47829. doi: 10.1097/MD.0000000000047829 (PMC12956227; doi:10.1097/MD.0000000000047829)
Supplement: Supplementary file 1 [file medi-105-e47829-s001.docx]

Supplementary Figure 1. Relative abundance of immune-cell subsets inferred by CIBERSORT in the LGG tumor micro-environment.


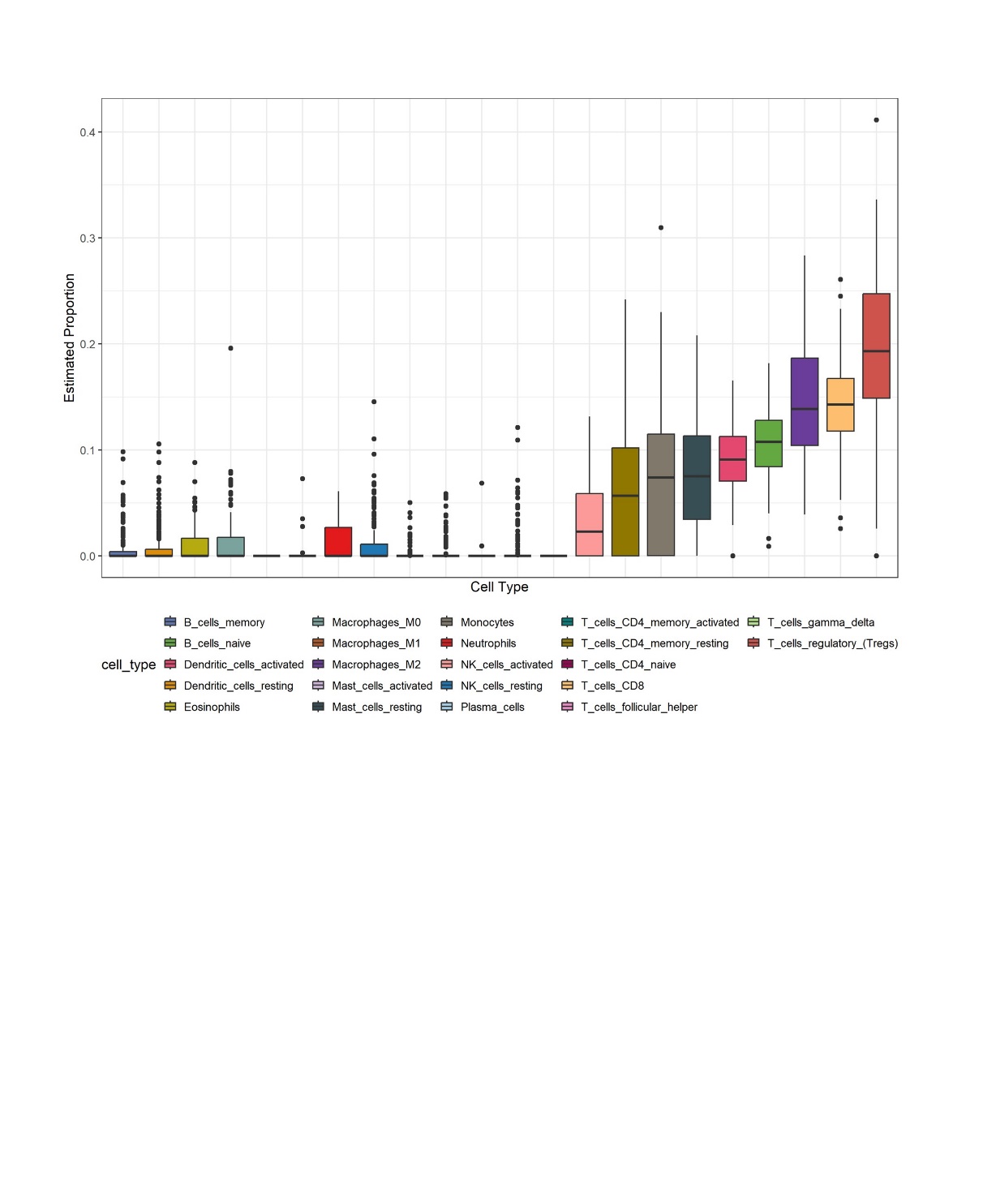


Supplementary Figure 2. Forest plot of hazard ratios (HRs) for metabolic and immune features associated with overall survival. Horizontal bars, 95 % CI; vertical line, null effect (HR = 1).


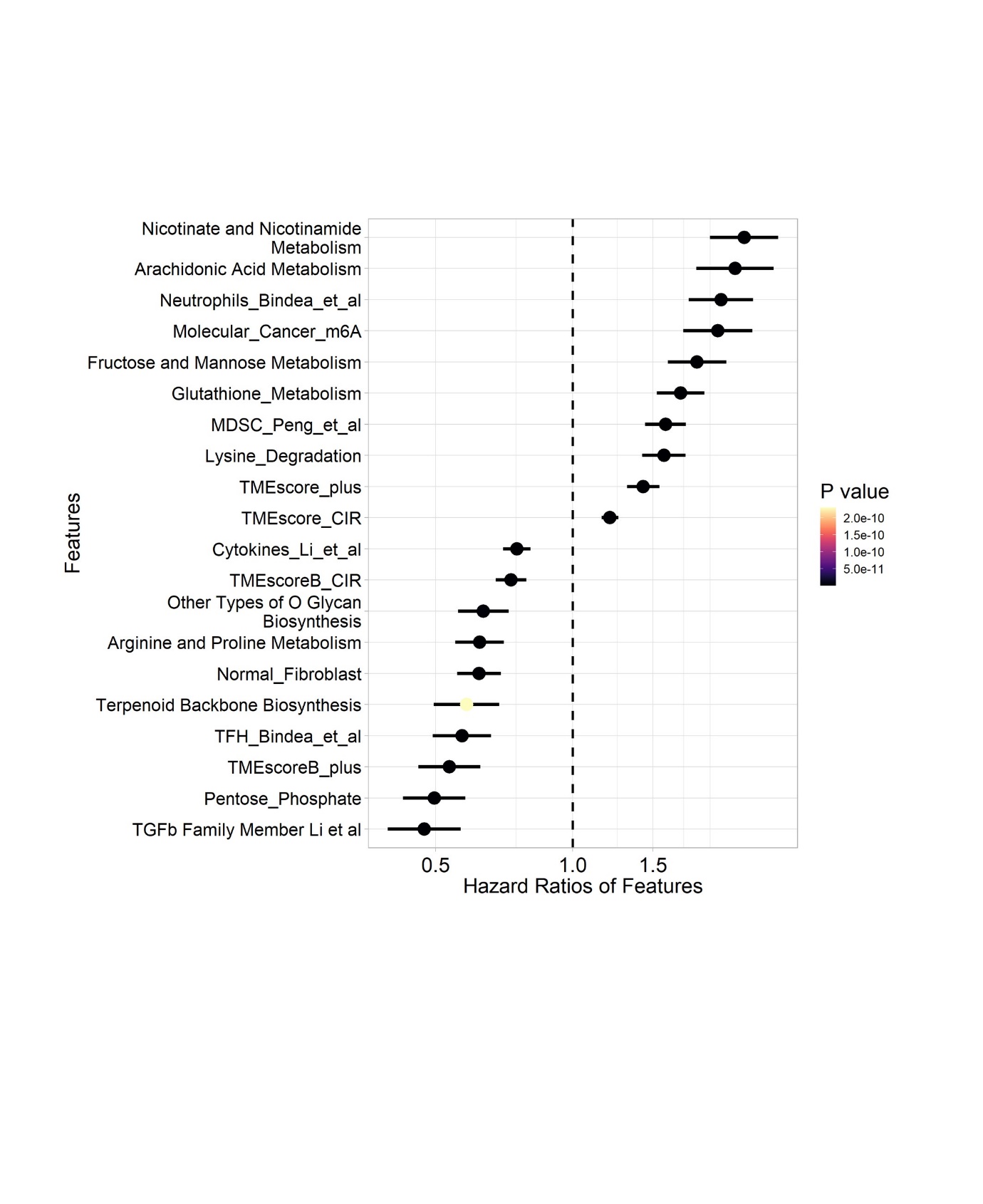


Supplementary Figure 3. Forest plot of HRs for metabolic and immune features associated with relapse-free survival. Horizontal bars, 95 % CI; vertical line, null effect (HR = 1).


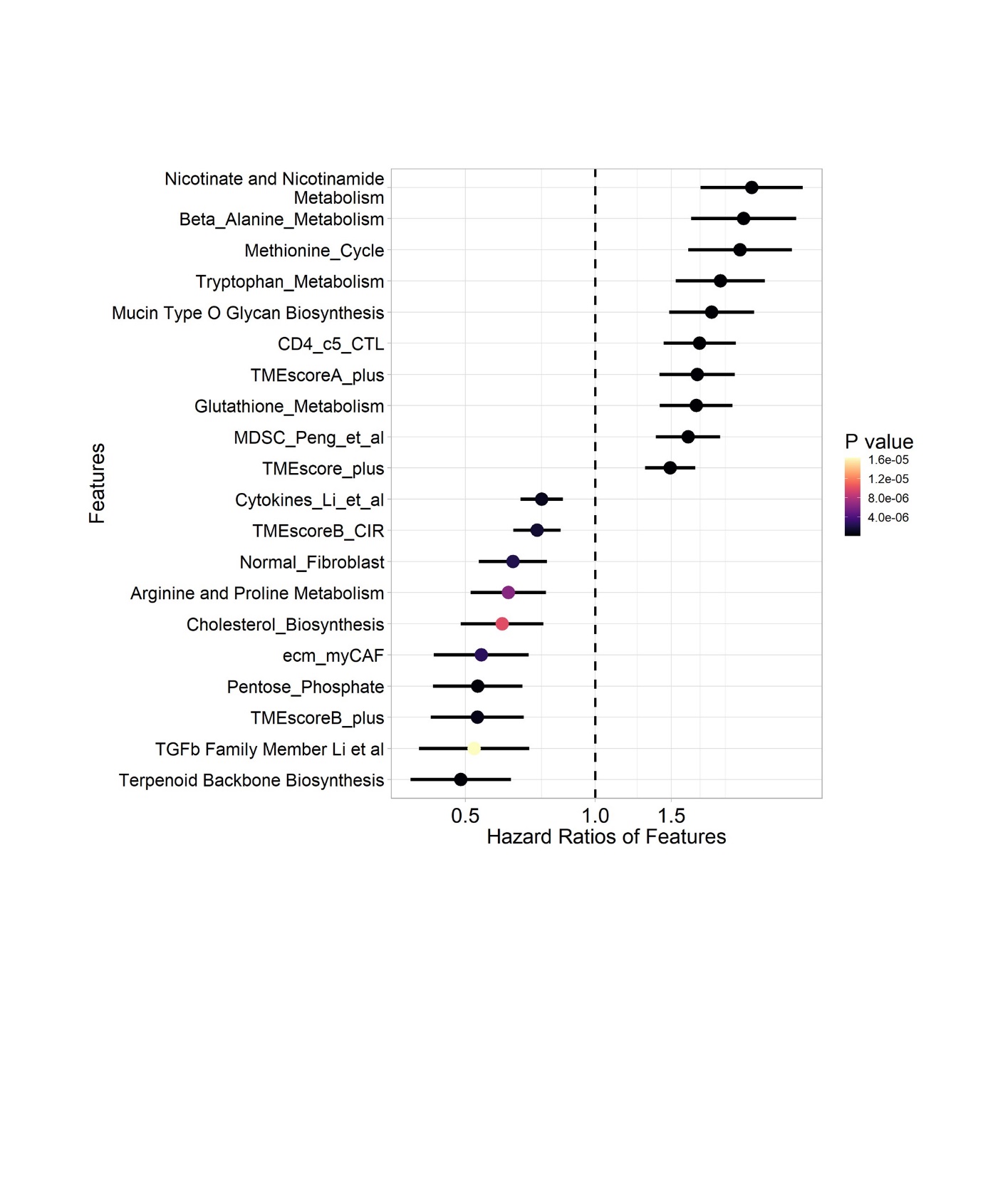


Supplementary Figure 4. Immune-cell composition across three TME clusters. (A) Heat-map showing distinct immune-cell signatures defining clusters TME1–TME3. (B, C) Box-plots of 2 leukocyte subsets, monocytes and activated memory CD4⁺ T-cell fractions across clusters.


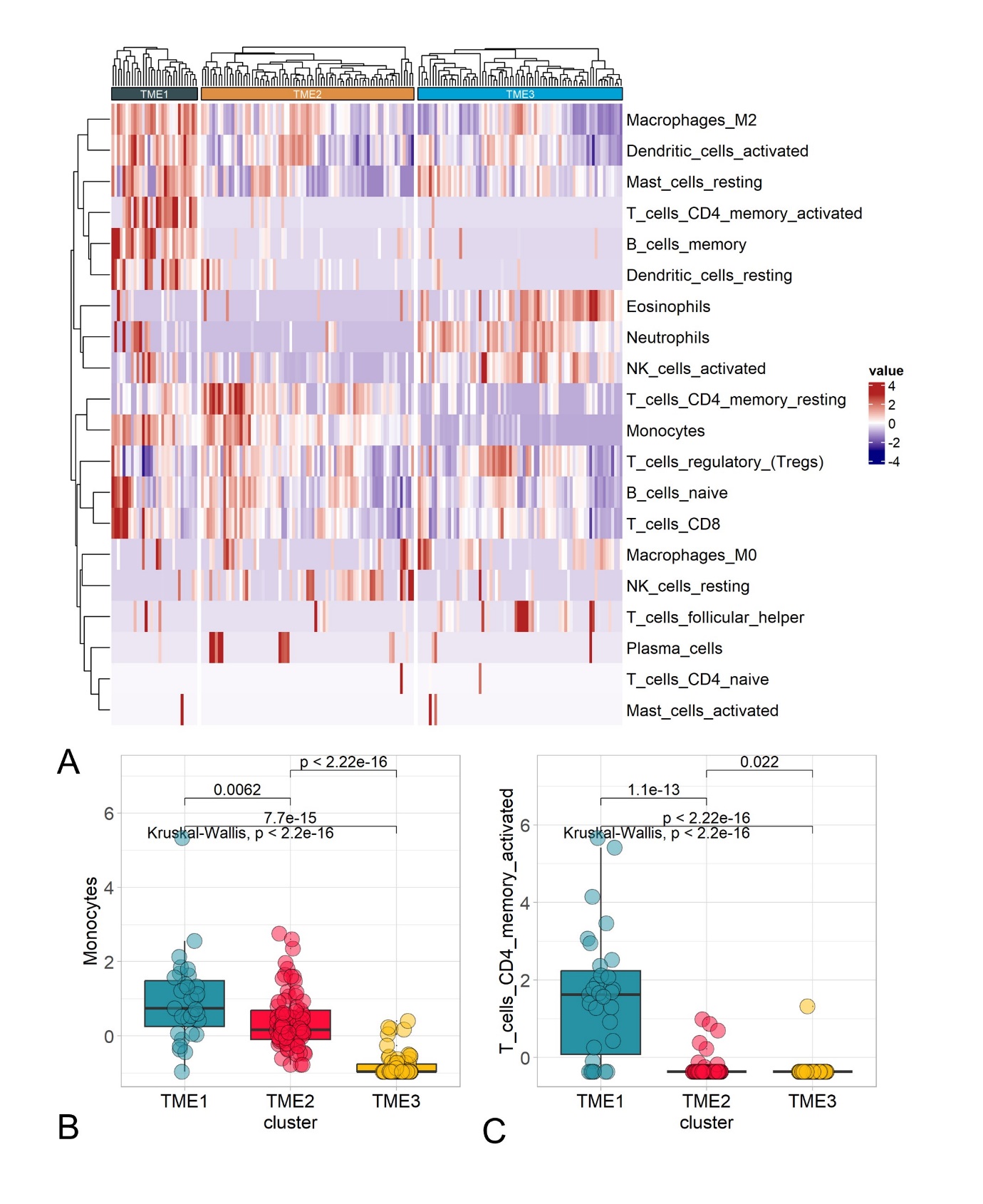


Supplementary Figure 5. Differential gene expression across TME clusters. (A) Heat-map of differentially expressed genes . (B, C) Box-plots of CD68 and ARHGDIB expression in TME1–TME3.


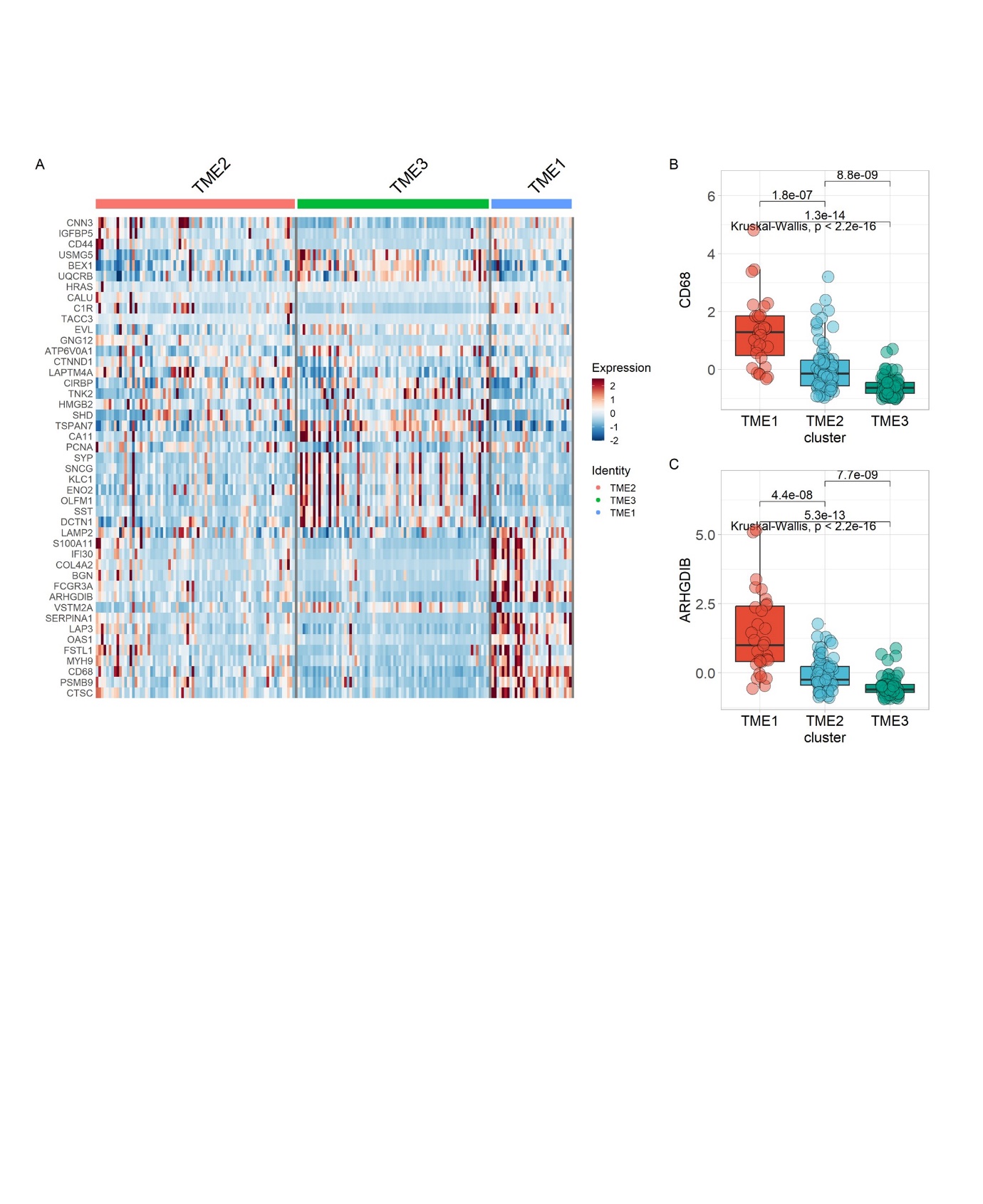


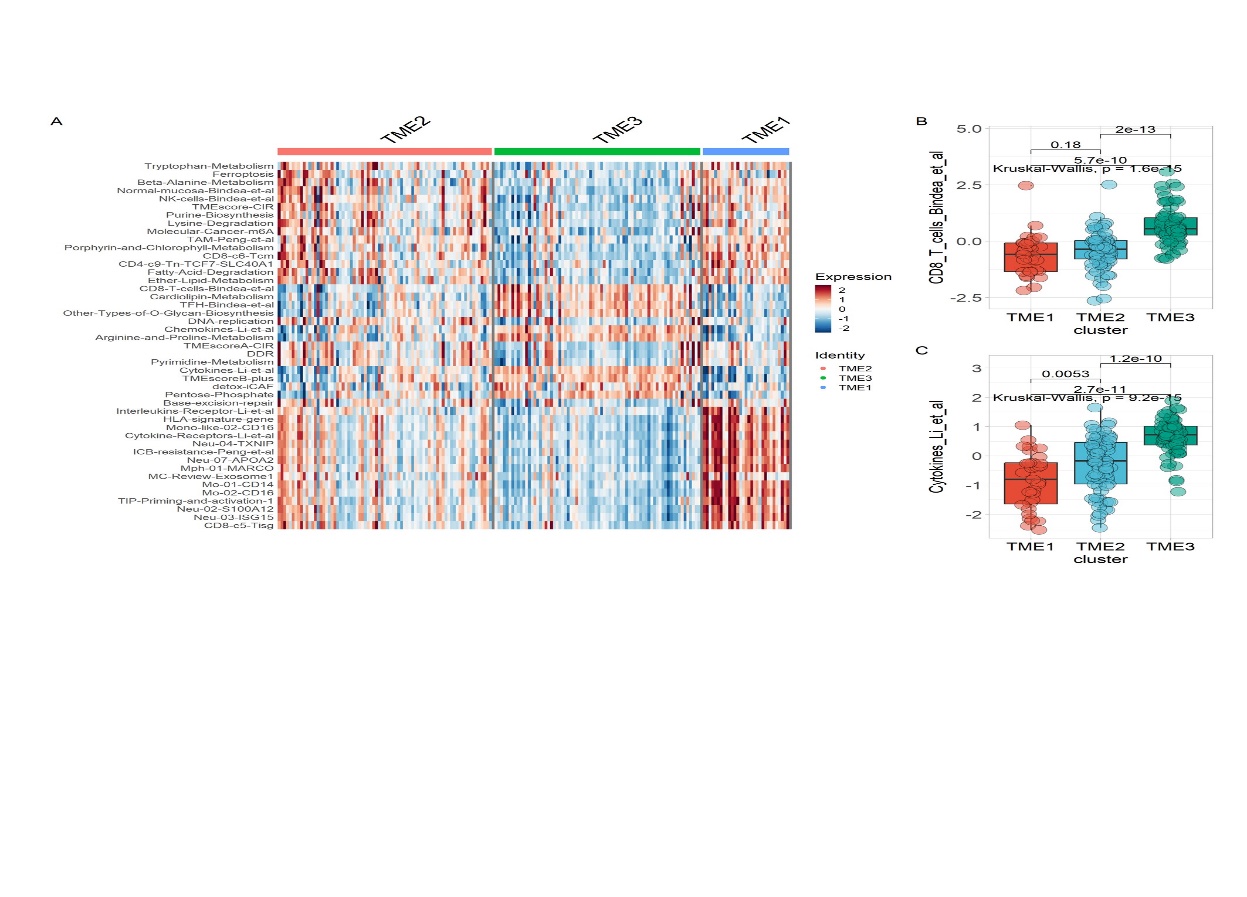


Supplementary Figure 6. Signature scores across TME clusters. (A) Heat-map of signature scores. (B, C) Box-plots of CD8_T_cells_Bindea_et_al and Cytokines_Li_et_al scores in TME1–TME3.


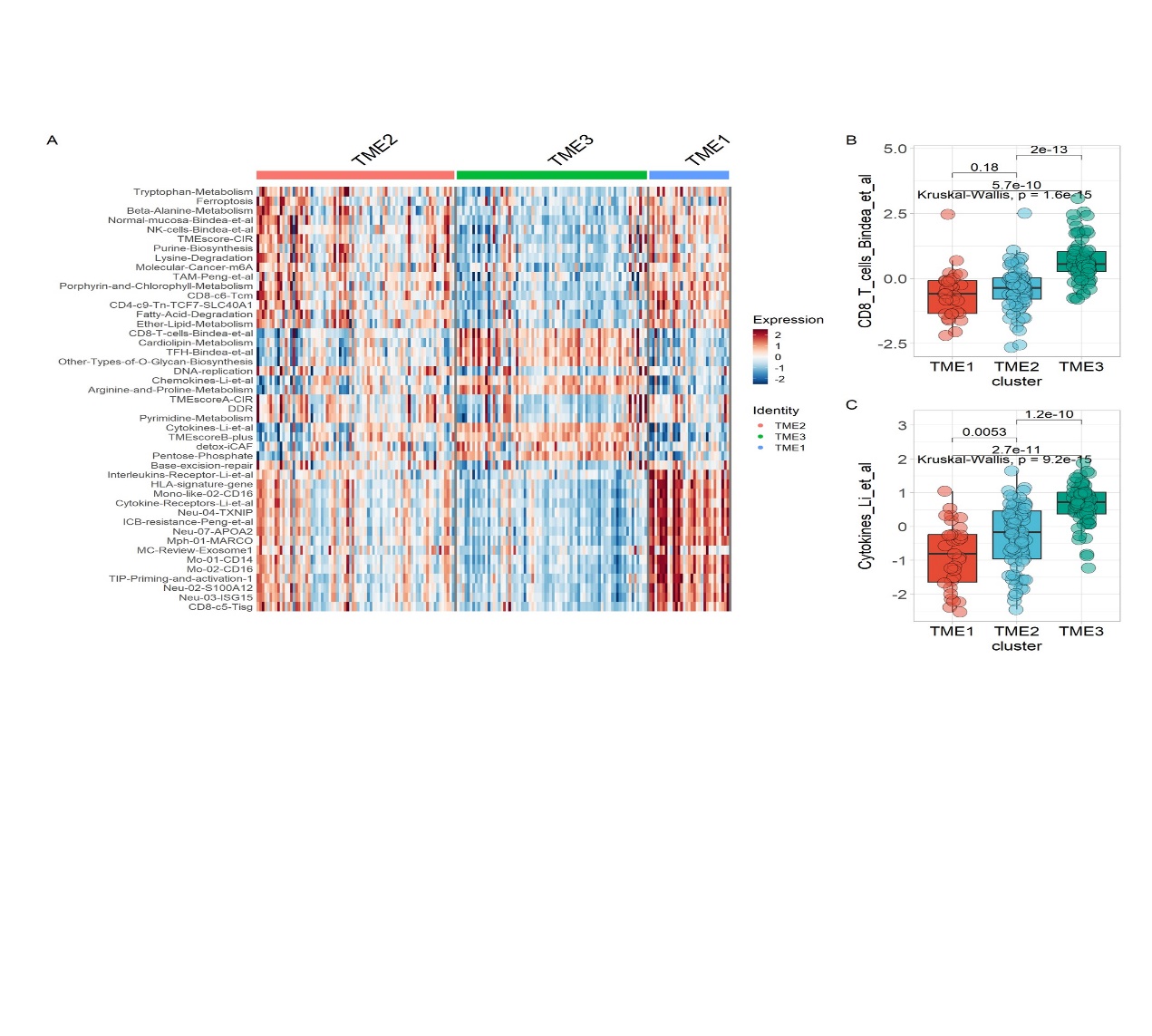


Supplementary Figure 7. Kaplan–Meier curves for overall survival of LGG patients stratified by TME clusters 1–3


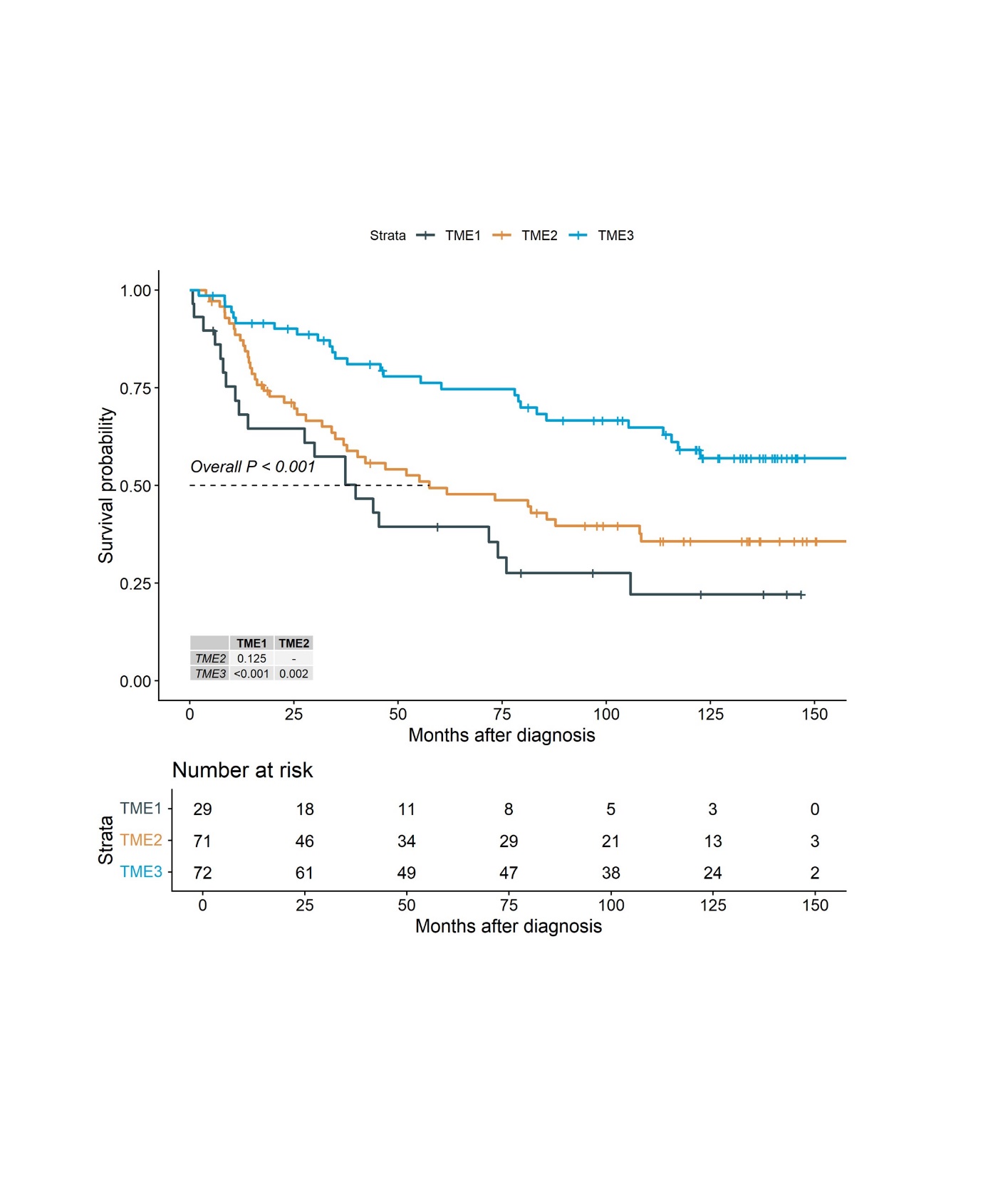


Supplementary Figure 8. Kaplan–Meier curves for relapse-free survival of LGG patients stratified by TME clusters 1–3


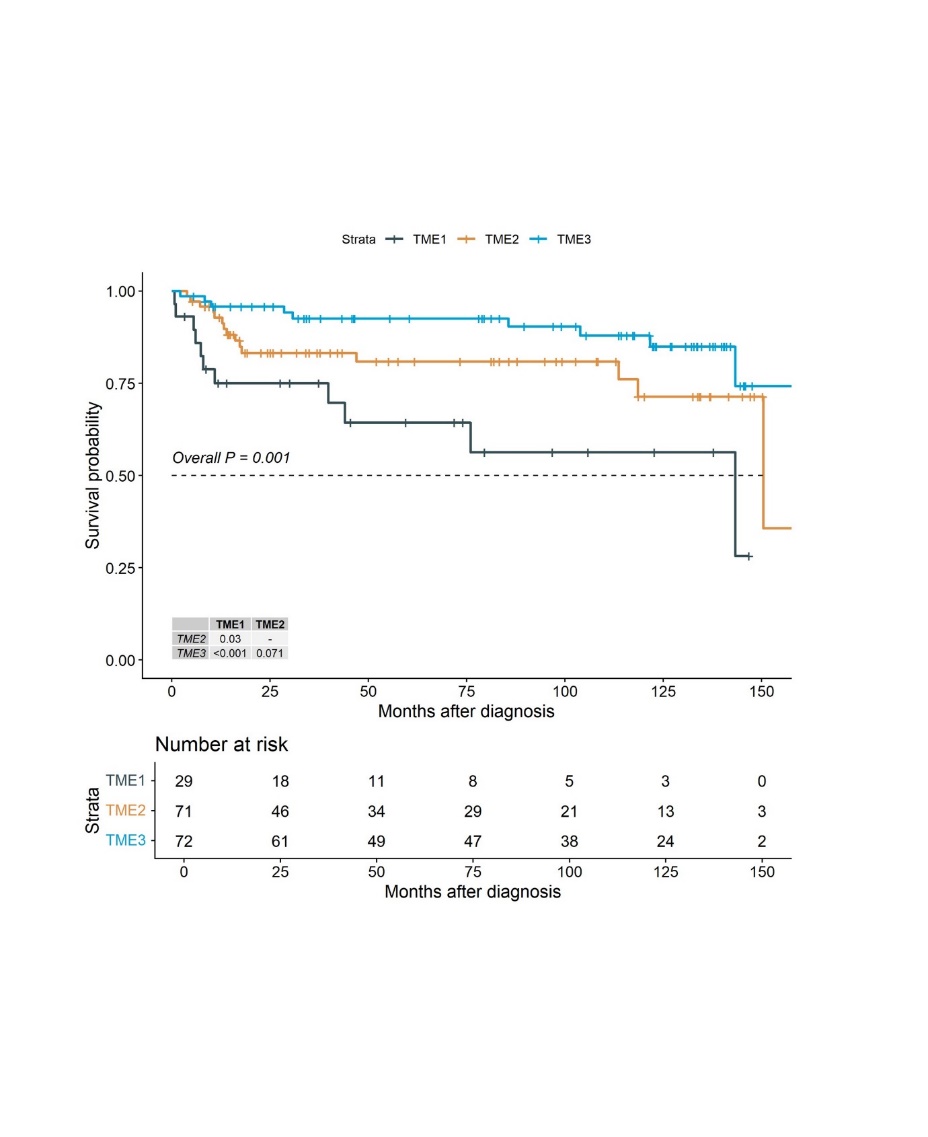


Supplementary Figure 9. High 71-gene score predicts shorter relapse-free survival. (A) 71-gene scores in relapsed vs non-relapsed patients (TCGA and CGGA). (B, C) Kaplan–Meier RFS curves by high vs low 71-gene score in TCGA and CGGA. Risk score = 71-gene score.


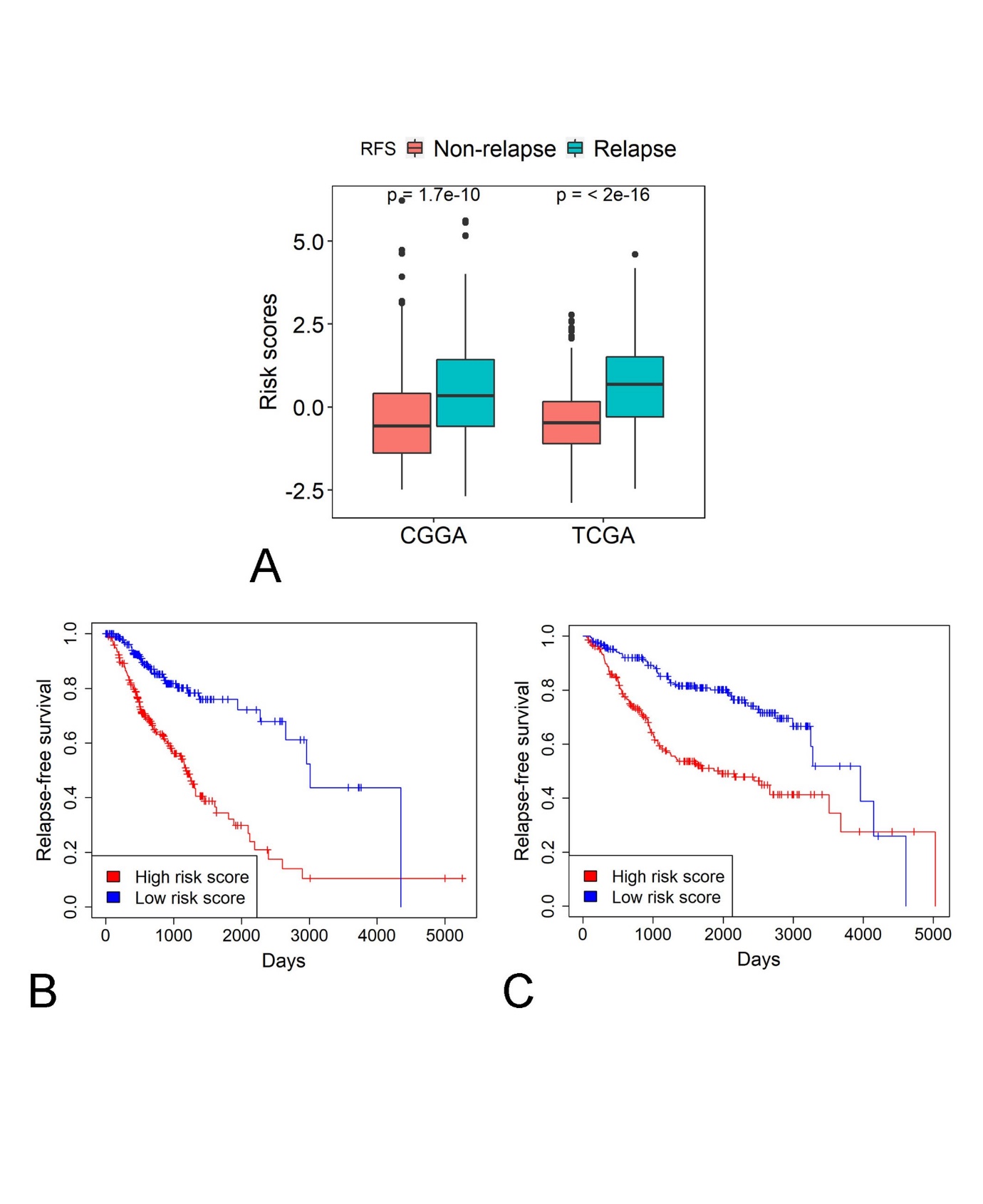


Supplementary Figure 10. OS by 71-gene score within TCGA sub-groups defined by age, sex and histological grade (A–F).


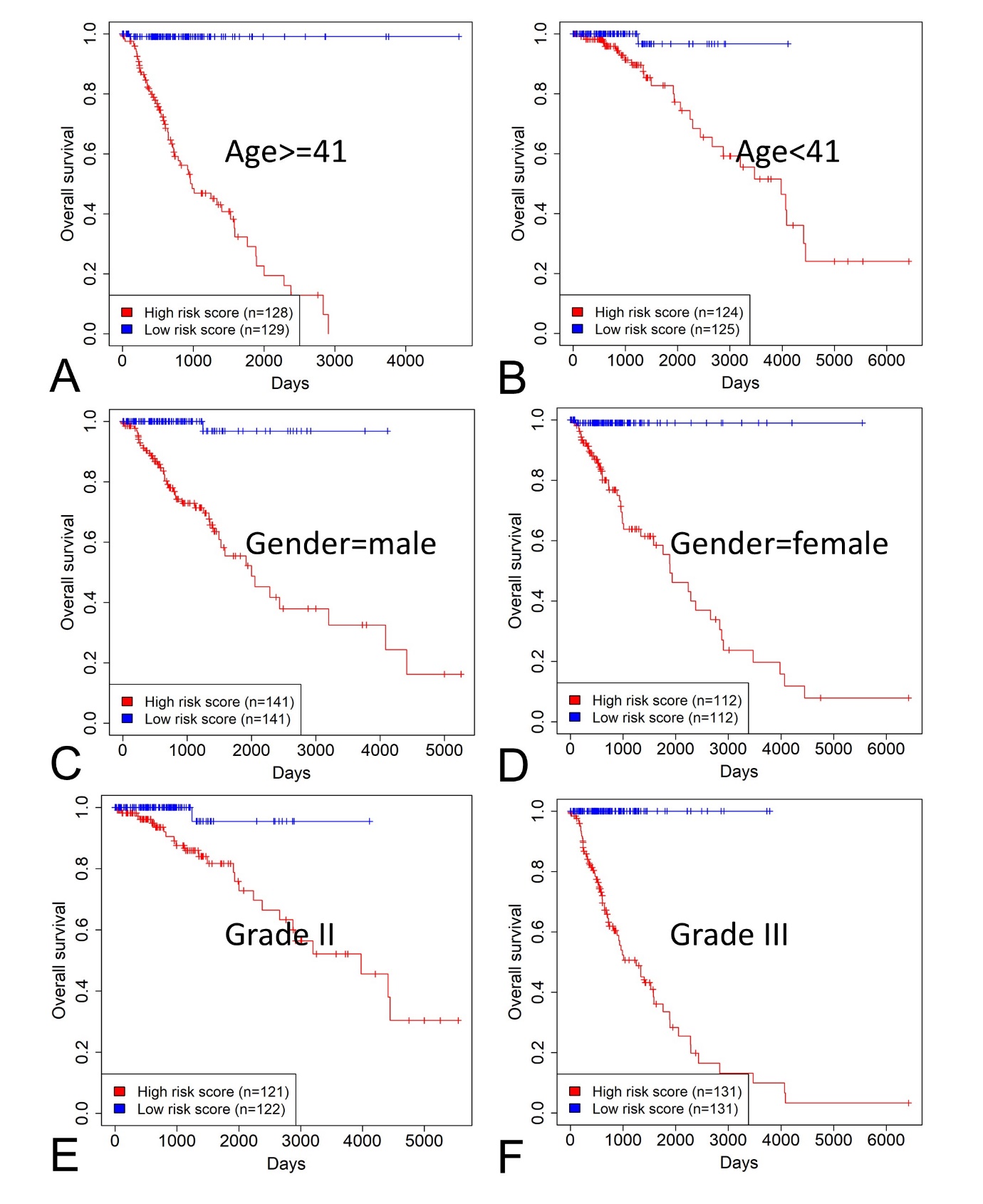


Supplementary Figure 11. OS by 71-gene score within TCGA sub-groups defined by cancer history, IDH1 mutation and radiotherapy (A–F).


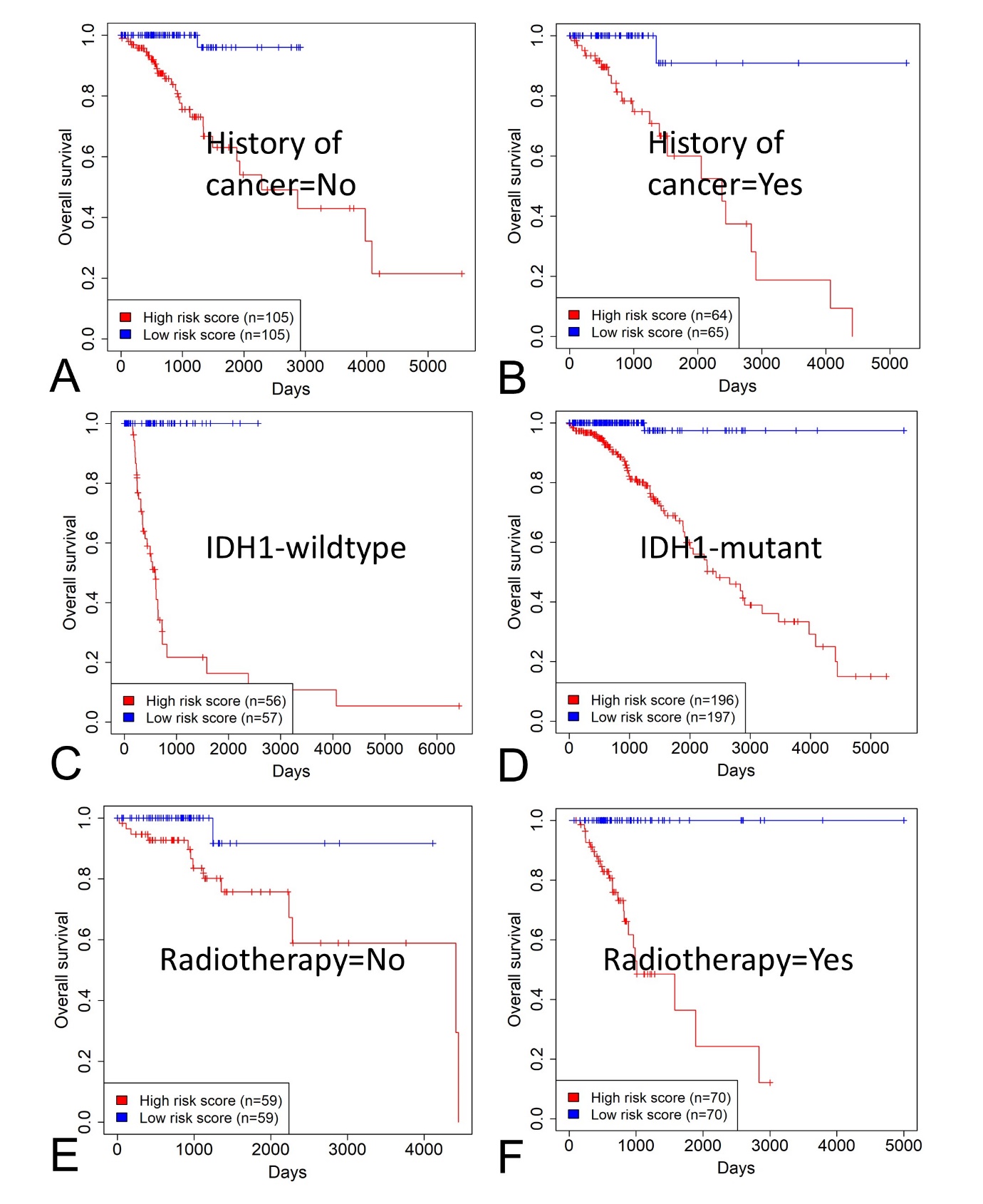


Supplementary Figure 12. OS by 71-gene score within TCGA sub-groups defined by targeted therapy, TP53 mutation and tumor weight (A–F).


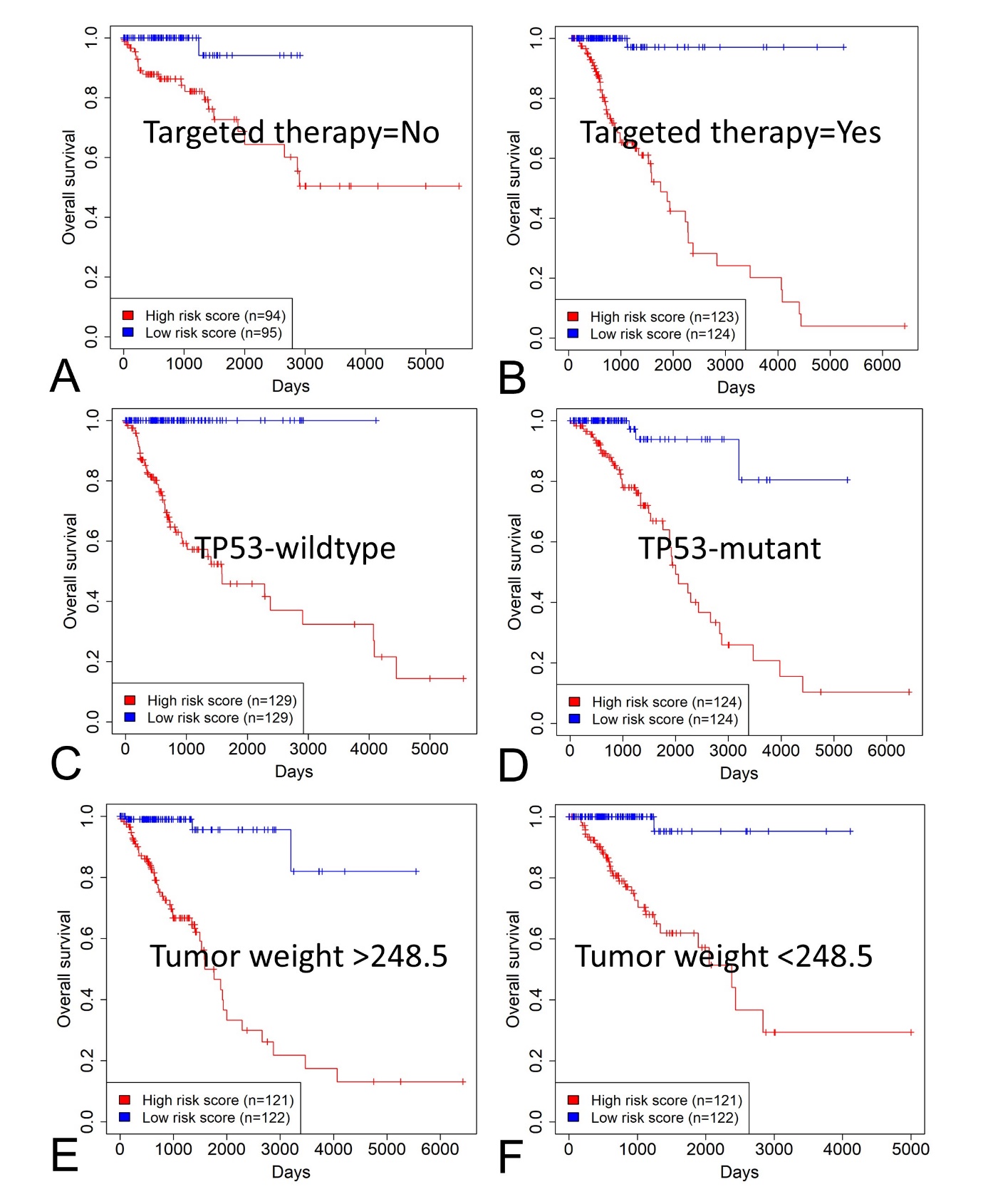


Supplementary Figure 13. OS by 71-gene score within CGGA sub-groups defined by age, sex and histological grade (A–F).


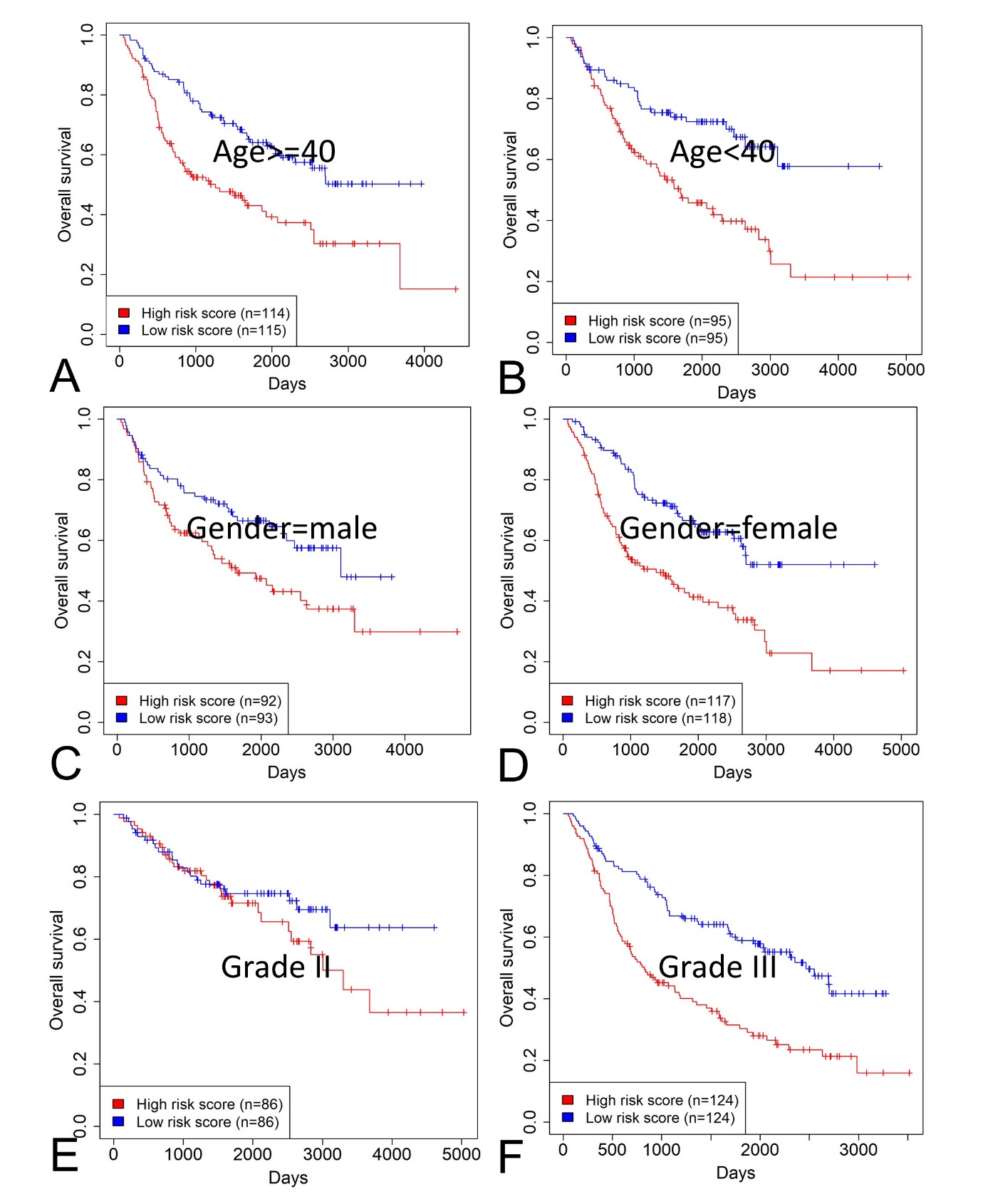


Supplementary Figure 14. OS by 71-gene score within CGGA sub-groups defined by IDH1 mutation, 1p/19q co-deletion and MGMT promoter methylation (A–F).


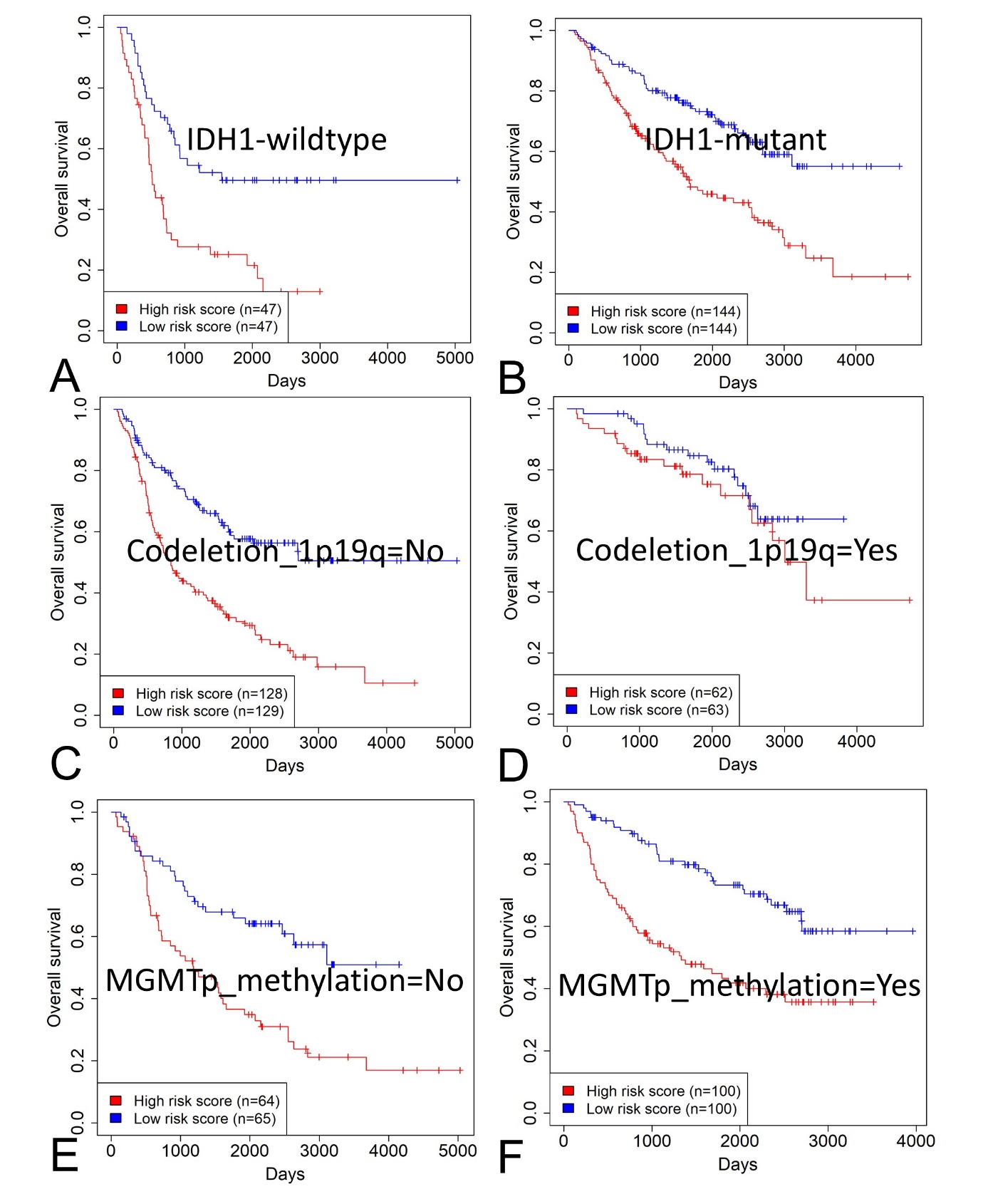


Supplementary Figure 15. OS by 71-gene score within CGGA sub-groups defined by radiotherapy and chemotherapy (A–D).


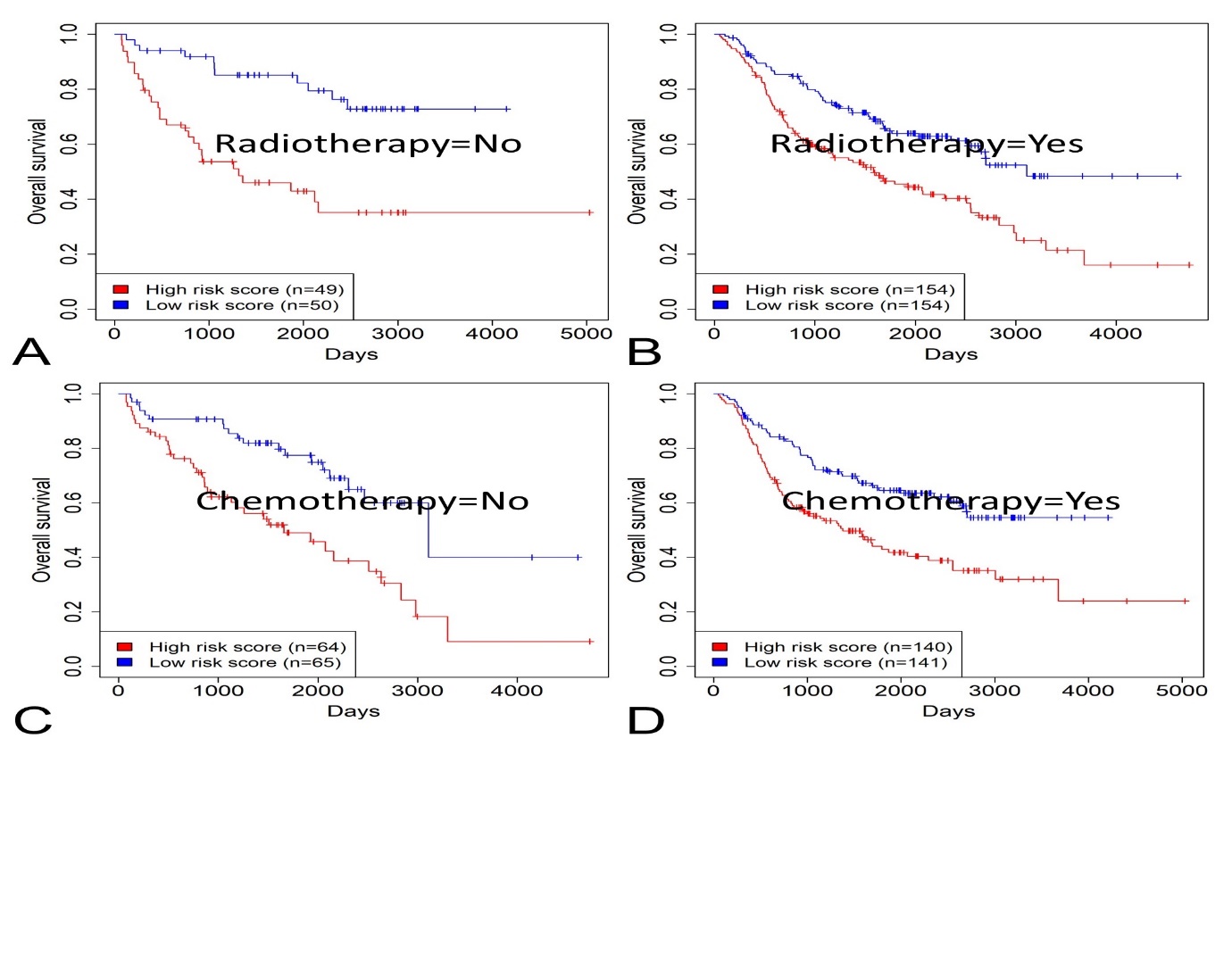


Supplementary Figure 16. RFS by 71-gene score in TCGA (A) and CGGA (B) data sets.


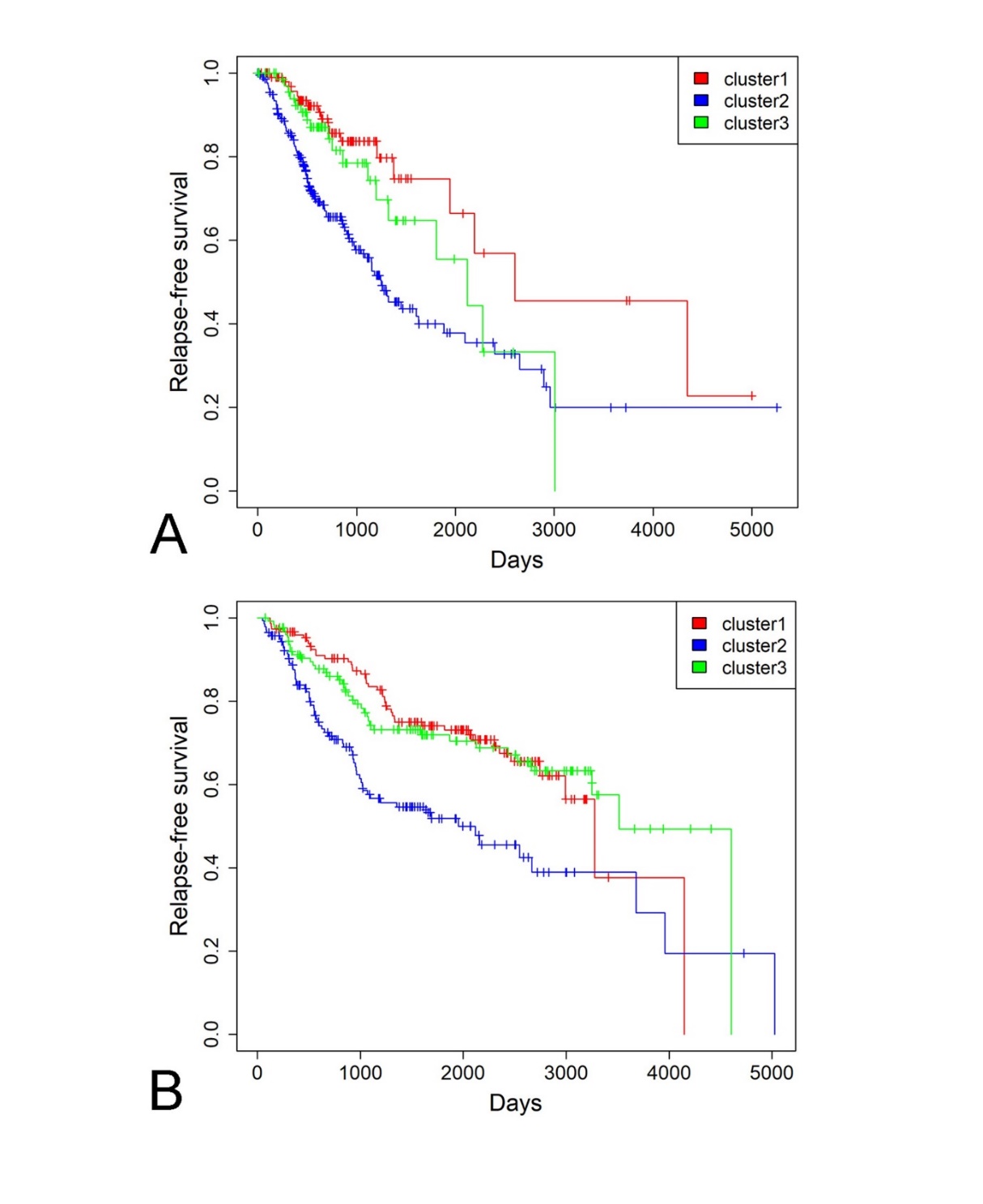


Supplementary Figure 17. 71-gene nomogram for relapse-free survival. (A) Nomogram incorporating histological grade (2 = grade II, 3 = grade III) and 71-gene score (high vs low, median split). (B) ROC curves for RFS prediction in TCGA and CGGA. (C, D) ROC curves for 3- and 5-year RFS in TCGA (C) and CGGA (D).


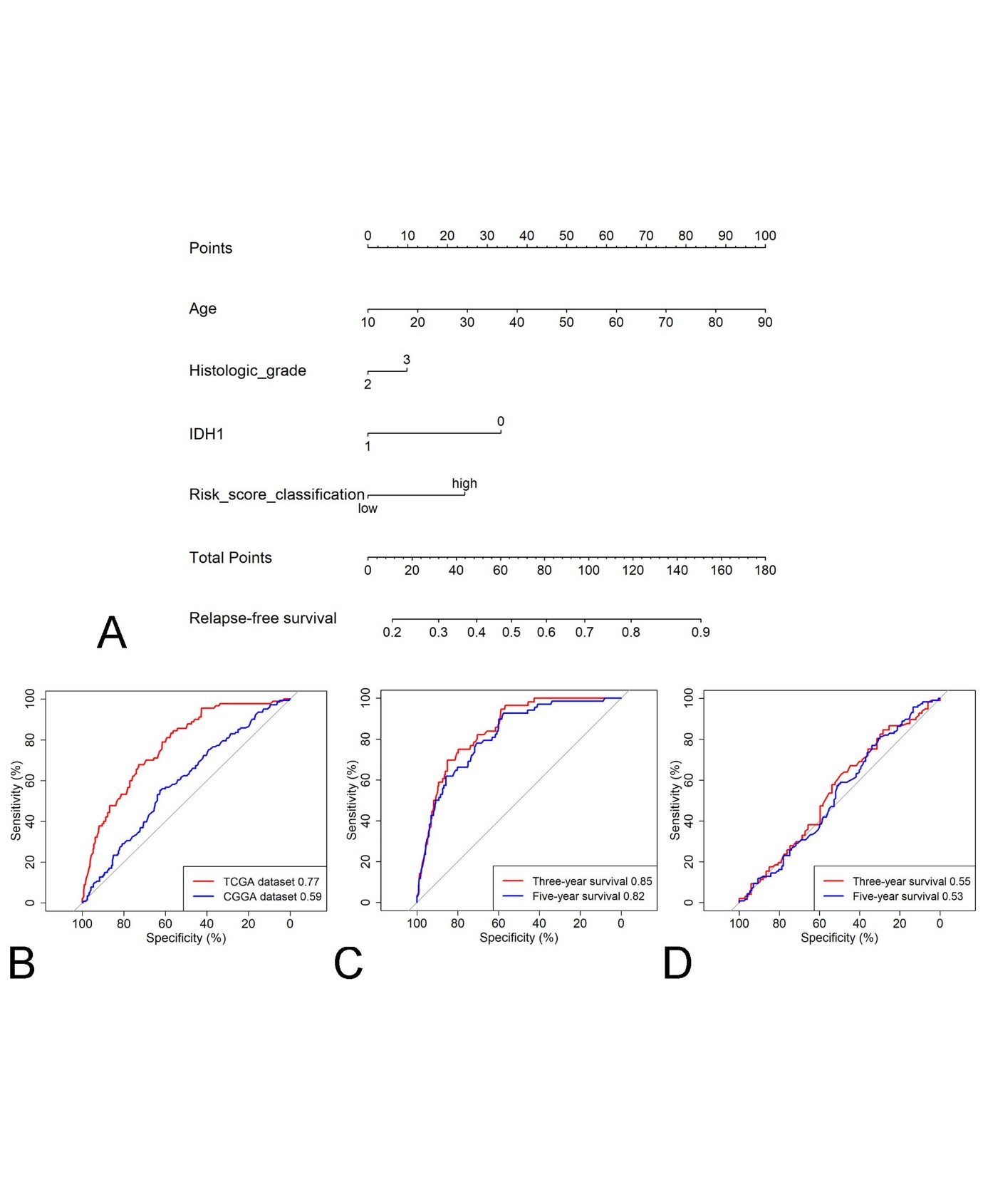


Supplementary Table1. Association between the clinicopathologic characteristics and patients’ mortality in the CGGA dataset

| Variables | Group | Alive | Dead | P value | Non-relapse | Relapse | P value | Statistical method |
| --- | --- | --- | --- | --- | --- | --- | --- | --- |
| Age |  | 39.68 | 40.93 | 0.21 | 39.98 | 40.51 | 0.59 | Student t test |
| Gender | Female | 103 | 83 | 0.43 | 124 | 69 | 0.84 | Fisher’s exact test |
|  | Male | 123 | 116 |  | 158 | 93 |  |  |
| Grade | II | 122 | 54 | <0.001 | 138 | 50 | <0.001 | Fisher’s exact test |
|  | III | 104 | 145 |  | 144 | 111 |  |  |
| *IDH1* mutation | Wild-type | 34 | 60 | <0.001 | 66 | 30 | 0.15 | Fisher’s exact test |
|  | Mutant | 165 | 128 |  | 184 | 123 |  |  |
| 1p19q_codeletion | Wild-type  Mutant | 115  91 | 145  36 | <0.001 | 166  83 | 107  49 | 0.74 | Fisher’s exact test |
| MGMTp methylation | No | 57 | 72 | 0.04 | 89 | 40 | 0.34 | Fisher’s exact test |
|  | Yes | 112 | 88 |  | 127 | 73 |  |  |
| Radiation therapy | No | 60 | 39 | 0.16 | 67 | 35 | 0.91 | Fisher’s exact test |
|  | Yes | 163 | 150 |  | 203 | 112 |  |  |
| Chemotherapy | No | 75 | 56 | 0.4 | 95 | 37 | 0.02 | Fisher’s exact test |
|  | Yes | 148 | 135 |  | 172 | 113 |  |  |

Supplementary Table2. The associations of 71 genes with overall survival of LGG patients in the TCGA dataset

|  | Kaplan-Meier overall analysis | | | Multivariate analysis | | | |
| --- | --- | --- | --- | --- | --- | --- | --- |
|  | Chisq value | P value | Median | HR | 2.50%CI | 97.50%CI | P value |
| ALG6 | 29.01 | <0.001 | 155.29 | 2.43 | 1.28 | 4.61 | 0.01 |
| APEX2 | 16.46 | <0.001 | 314.74 | 1.88 | 1.05 | 3.39 | 0.03 |
| AQP7 | 29.22 | <0.001 | 2.09 | 0.23 | 0.12 | 0.43 | <0.001 |
| BCORL1 | 11.03 | <0.001 | 299.51 | 2.09 | 1.19 | 3.68 | 0.01 |
| BST1 | 16.55 | <0.001 | 23.13 | 2.50 | 1.39 | 4.51 | <0.001 |
| CA4 | 6.95 | 0.01 | 156.18 | 0.51 | 0.30 | 0.88 | 0.02 |
| CARHSP1 | 20.52 | <0.001 | 940.73 | 2.14 | 1.20 | 3.80 | 0.01 |
| CCT6A | 10.40 | <0.001 | 3060.71 | 1.95 | 1.06 | 3.60 | 0.03 |
| CDH22 | 7.85 | 0.01 | 238.00 | 0.50 | 0.29 | 0.87 | 0.01 |
| CMTM1 | 8.67 | <0.001 | 139.81 | 2.33 | 1.32 | 4.11 | <0.001 |
| CNOT7 | 4.93 | 0.03 | 1165.88 | 1.81 | 1.06 | 3.08 | 0.03 |
| COPA | 14.64 | <0.001 | 4820.13 | 2.30 | 1.27 | 4.19 | 0.01 |
| COX16 | 8.84 | <0.001 | 818.61 | 0.56 | 0.32 | 0.97 | 0.04 |
| DDX25 | 15.99 | <0.001 | 336.55 | 0.50 | 0.28 | 0.89 | 0.02 |
| EIF3F | 9.37 | <0.001 | 2040.18 | 0.56 | 0.32 | 0.98 | 0.04 |
| FASTKD5 | 7.48 | 0.01 | 358.47 | 2.92 | 1.66 | 5.13 | <0.001 |
| FMO5 | 8.10 | <0.001 | 47.90 | 2.47 | 1.45 | 4.19 | <0.001 |
| GBP5 | 11.73 | <0.001 | 13.53 | 1.77 | 1.01 | 3.10 | 0.05 |
| GDF10 | 16.36 | <0.001 | 96.64 | 0.42 | 0.23 | 0.75 | <0.001 |
| GIT1 | 4.46 | 0.03 | 2927.72 | 0.47 | 0.28 | 0.80 | 0.01 |
| GLG1 | 4.46 | 0.03 | 5285.37 | 2.27 | 1.33 | 3.89 | <0.001 |
| GRPR | 9.25 | <0.001 | 3.83 | 2.05 | 1.18 | 3.58 | 0.01 |
| HAUS1 | 9.68 | <0.001 | 217.86 | 2.31 | 1.30 | 4.09 | <0.001 |
| HIST1H3E | 21.06 | <0.001 | 10.25 | 2.71 | 1.44 | 5.12 | <0.001 |
| HIST2H4A | 12.10 | <0.001 | 157.17 | 2.91 | 1.60 | 5.29 | <0.001 |
| HLA.DQB1 | 9.07 | <0.001 | 254.70 | 2.34 | 1.35 | 4.06 | <0.001 |
| HSD17B7P2 | 11.25 | <0.001 | 55.88 | 0.56 | 0.32 | 0.99 | 0.05 |
| IL3RA | 6.18 | 0.01 | 69.23 | 0.53 | 0.31 | 0.92 | 0.03 |
| KCNK4 | 19.19 | <0.001 | 62.83 | 0.48 | 0.26 | 0.86 | 0.01 |
| KTI12 | 10.71 | <0.001 | 164.57 | 2.14 | 1.22 | 3.74 | 0.01 |
| LRRC16B | 12.82 | <0.001 | 323.93 | 0.41 | 0.23 | 0.73 | <0.001 |
| MBOAT4 | 12.10 | <0.001 | 2.22 | 2.10 | 1.23 | 3.60 | 0.01 |
| MEN1 | 17.76 | <0.001 | 850.49 | 2.24 | 1.22 | 4.13 | 0.01 |
| MRPL46 | 13.39 | <0.001 | 423.46 | 0.53 | 0.30 | 0.94 | 0.03 |
| MTIF2 | 14.75 | <0.001 | 512.45 | 2.18 | 1.24 | 3.83 | 0.01 |
| NAT6 | 5.01 | 0.03 | 221.45 | 0.42 | 0.23 | 0.75 | <0.001 |
| NUP107 | 14.38 | <0.001 | 463.87 | 2.07 | 1.14 | 3.75 | 0.02 |
| OAS1 | 15.75 | <0.001 | 157.89 | 1.78 | 1.03 | 3.06 | 0.04 |
| PACSIN2 | 6.48 | 0.01 | 845.56 | 1.80 | 1.04 | 3.09 | 0.03 |
| PALLD | 10.47 | <0.001 | 1618.09 | 2.02 | 1.14 | 3.61 | 0.02 |
| PHACTR4 | 9.37 | <0.001 | 827.52 | 2.56 | 1.43 | 4.57 | <0.001 |
| PROX1 | 3.86 | 0.05 | 134.96 | 2.01 | 1.15 | 3.51 | 0.01 |
| PTCRA | 22.12 | <0.001 | 2.72 | 2.68 | 1.54 | 4.66 | <0.001 |
| PTPRN | 14.18 | <0.001 | 1032.63 | 0.30 | 0.16 | 0.54 | <0.001 |
| RABGGTA | 8.58 | <0.001 | 356.93 | 0.31 | 0.18 | 0.55 | <0.001 |
| RAD18 | 23.31 | <0.001 | 111.52 | 2.46 | 1.32 | 4.58 | <0.001 |
| SAMD9 | 18.86 | <0.001 | 190.86 | 2.45 | 1.38 | 4.37 | <0.001 |
| SAP30 | 14.02 | <0.001 | 355.15 | 1.93 | 1.14 | 3.28 | 0.01 |
| SEC31A | 11.65 | <0.001 | 2074.86 | 2.06 | 1.11 | 3.82 | 0.02 |
| SERF1A | 6.22 | 0.01 | 873.20 | 0.51 | 0.30 | 0.86 | 0.01 |
| SERTAD2 | 12.83 | <0.001 | 499.09 | 1.82 | 1.05 | 3.15 | 0.03 |
| SKI | 18.81 | <0.001 | 2474.66 | 1.81 | 1.01 | 3.24 | 0.05 |
| SLC11A1 | 25.01 | <0.001 | 197.69 | 2.14 | 1.23 | 3.72 | 0.01 |
| SLC16A1 | 30.24 | <0.001 | 2065.12 | 2.91 | 1.61 | 5.27 | <0.001 |
| SLC25A13 | 15.39 | <0.001 | 442.63 | 2.09 | 1.21 | 3.59 | 0.01 |
| SLC26A2 | 18.29 | <0.001 | 245.34 | 2.04 | 1.09 | 3.80 | 0.02 |
| SOX9 | 13.60 | <0.001 | 3923.46 | 2.40 | 1.38 | 4.18 | <0.001 |
| SPATA6 | 13.82 | <0.001 | 112.70 | 2.67 | 1.45 | 4.93 | <0.001 |
| SULF2 | 31.55 | <0.001 | 6383.15 | 0.54 | 0.30 | 0.98 | 0.04 |
| TBX4 | 4.72 | 0.03 | 1.20 | 1.85 | 1.09 | 3.13 | 0.02 |
| TFPI | 11.46 | <0.001 | 128.61 | 2.36 | 1.34 | 4.18 | <0.001 |
| TGIF1 | 35.08 | <0.001 | 254.80 | 3.83 | 2.00 | 7.34 | <0.001 |
| TMEM71 | 33.35 | <0.001 | 9.81 | 2.00 | 1.10 | 3.62 | 0.02 |
| TMEM87B | 20.20 | <0.001 | 259.78 | 2.15 | 1.23 | 3.74 | 0.01 |
| TNK2 | 22.01 | <0.001 | 6325.26 | 0.44 | 0.24 | 0.82 | 0.01 |
| TNNI3 | 5.09 | 0.02 | 3.05 | 0.49 | 0.29 | 0.83 | 0.01 |
| TP73 | 17.79 | <0.001 | 12.01 | 1.88 | 1.07 | 3.33 | 0.03 |
| TSPYL2 | 8.14 | <0.001 | 2282.08 | 0.45 | 0.25 | 0.79 | 0.01 |
| TULP3 | 14.42 | <0.001 | 492.91 | 2.69 | 1.48 | 4.90 | <0.001 |
| WNT7B | 11.73 | <0.001 | 404.06 | 0.52 | 0.29 | 0.93 | 0.03 |
| ZCCHC4 | 9.79 | <0.001 | 140.53 | 2.29 | 1.34 | 3.91 | <0.001 |

Supplementary Table3. The associations of 71 genes with overall survival of LGG patients in the CGGA dataset

|  | Kaplan-Meier overall analysis | | | Multivariate analysis | | | |
| --- | --- | --- | --- | --- | --- | --- | --- |
|  | Chisq value | P value | Median | HR | 2.50%CI | 97.50%CI | P value |
| ALG6 | 15.95 | <0.001 | 3.78 | 1.49 | 1.07 | 2.06 | 0.02 |
| APEX2 | 14.53 | <0.001 | 5.36 | 1.50 | 1.09 | 2.07 | 0.01 |
| AQP7 | 18.04 | <0.001 | 0.16 | 0.65 | 0.47 | 0.89 | 0.01 |
| BCORL1 | 14.80 | <0.001 | 1.58 | 1.44 | 1.04 | 1.99 | 0.03 |
| BST1 | 28.76 | <0.001 | 1.24 | 2.04 | 1.45 | 2.86 | <0.001 |
| CA4 | 16.72 | <0.001 | 9.64 | 0.61 | 0.44 | 0.83 | <0.001 |
| CARHSP1 | 8.94 | <0.001 | 24.10 | 1.39 | 1.01 | 1.92 | 0.04 |
| CCT6A | 19.25 | <0.001 | 69.24 | 1.73 | 1.23 | 2.43 | <0.001 |
| CDH22 | 24.54 | <0.001 | 6.24 | 0.58 | 0.42 | 0.80 | <0.001 |
| CMTM1 | 12.43 | <0.001 | 2.11 | 1.61 | 1.17 | 2.22 | <0.001 |
| CNOT7 | 6.14 | 0.01 | 13.89 | 1.52 | 1.11 | 2.09 | 0.01 |
| COPA | 8.73 | <0.001 | 29.82 | 1.63 | 1.19 | 2.25 | <0.001 |
| COX16 | 17.56 | <0.001 | 37.74 | 0.67 | 0.49 | 0.92 | 0.01 |
| DDX25 | 10.47 | <0.001 | 10.57 | 0.57 | 0.42 | 0.78 | <0.001 |
| EIF3F | 10.67 | <0.001 | 103.63 | 0.67 | 0.48 | 0.94 | 0.02 |
| FASTKD5 | 10.77 | <0.001 | 3.58 | 1.66 | 1.20 | 2.29 | <0.001 |
| FMO5 | 4.11 | 0.04 | 1.02 | 1.41 | 1.03 | 1.93 | 0.03 |
| GBP5 | 39.20 | <0.001 | 0.21 | 1.87 | 1.33 | 2.63 | <0.001 |
| GDF10 | 9.14 | <0.001 | 1.24 | 0.55 | 0.40 | 0.75 | <0.001 |
| GIT1 | 5.26 | 0.02 | 46.35 | 0.72 | 0.53 | 0.98 | 0.04 |
| GLG1 | 12.28 | <0.001 | 22.73 | 1.53 | 1.10 | 2.12 | 0.01 |
| GRPR | 16.44 | <0.001 | 0.02 | 1.76 | 1.29 | 2.39 | <0.001 |
| HAUS1 | 21.30 | <0.001 | 10.51 | 1.82 | 1.30 | 2.54 | <0.001 |
| HIST1H3E | 27.76 | <0.001 | 0.23 | 1.73 | 1.25 | 2.38 | <0.001 |
| HIST2H4A | 22.38 | <0.001 | 5.17 | 2.04 | 1.49 | 2.81 | <0.001 |
| HLA.DQB1 | 7.58 | 0.01 | 3.18 | 1.41 | 1.03 | 1.92 | 0.03 |
| HSD17B7P2 | 8.02 | <0.001 | 3.70 | 0.71 | 0.51 | 0.98 | 0.04 |
| IL3RA | 4.78 | 0.03 | 1.01 | 0.69 | 0.51 | 0.94 | 0.02 |
| KCNK4 | 20.59 | <0.001 | 2.69 | 0.52 | 0.38 | 0.71 | <0.001 |
| KTI12 | 42.16 | <0.001 | 4.56 | 2.07 | 1.43 | 3.00 | <0.001 |
| LRRC16B | 14.54 | <0.001 | 6.83 | 0.59 | 0.43 | 0.81 | <0.001 |
| MBOAT4 | 6.19 | 0.01 | 0.05 | 1.49 | 1.10 | 2.04 | 0.01 |
| MEN1 | 21.75 | <0.001 | 16.85 | 1.64 | 1.18 | 2.29 | <0.001 |
| MRPL46 | 9.57 | <0.001 | 22.78 | 0.72 | 0.52 | 0.98 | 0.04 |
| MTIF2 | 4.19 | 0.04 | 8.19 | 1.46 | 1.07 | 2.01 | 0.02 |
| NAT6 | 7.07 | 0.01 | 11.90 | 0.64 | 0.47 | 0.87 | <0.001 |
| NUP107 | 17.56 | <0.001 | 7.39 | 1.83 | 1.32 | 2.54 | <0.001 |
| OAS1 | 18.49 | <0.001 | 4.70 | 1.68 | 1.21 | 2.35 | <0.001 |
| PACSIN2 | 7.66 | 0.01 | 18.06 | 1.42 | 1.04 | 1.95 | 0.03 |
| PALLD | 9.33 | <0.001 | 12.36 | 1.55 | 1.12 | 2.15 | 0.01 |
| PHACTR4 | 23.48 | <0.001 | 3.43 | 1.75 | 1.23 | 2.50 | <0.001 |
| PROX1 | 11.47 | <0.001 | 1.55 | 1.64 | 1.20 | 2.25 | <0.001 |
| PTCRA | 19.34 | <0.001 | 0.29 | 1.49 | 1.08 | 2.06 | 0.01 |
| PTPRN | 14.24 | <0.001 | 30.82 | 0.69 | 0.50 | 0.94 | 0.02 |
| RABGGTA | 4.65 | 0.03 | 19.59 | 0.70 | 0.52 | 0.95 | 0.02 |
| RAD18 | 18.55 | <0.001 | 1.26 | 1.69 | 1.22 | 2.35 | <0.001 |
| SAMD9 | 13.61 | <0.001 | 0.70 | 1.68 | 1.21 | 2.33 | <0.001 |
| SAP30 | 16.86 | <0.001 | 18.51 | 1.57 | 1.11 | 2.20 | 0.01 |
| SEC31A | 12.04 | <0.001 | 24.65 | 1.66 | 1.20 | 2.30 | <0.001 |
| SERF1A | 5.60 | 0.02 | 8.24 | 0.69 | 0.50 | 0.94 | 0.02 |
| SERTAD2 | 4.37 | 0.04 | 4.39 | 1.55 | 1.13 | 2.13 | 0.01 |
| SKI | 7.76 | 0.01 | 11.97 | 1.41 | 1.03 | 1.94 | 0.03 |
| SLC11A1 | 30.48 | <0.001 | 5.16 | 1.58 | 1.12 | 2.23 | 0.01 |
| SLC16A1 | 24.45 | <0.001 | 13.07 | 2.14 | 1.52 | 3.02 | <0.001 |
| SLC25A13 | 16.32 | <0.001 | 6.67 | 1.86 | 1.33 | 2.59 | <0.001 |
| SLC26A2 | 17.41 | <0.001 | 0.98 | 1.75 | 1.27 | 2.42 | <0.001 |
| SOX9 | 11.61 | <0.001 | 26.10 | 1.79 | 1.29 | 2.49 | <0.001 |
| SPATA6 | 14.53 | <0.001 | 2.88 | 1.42 | 1.02 | 1.98 | 0.04 |
| SULF2 | 9.83 | <0.001 | 102.21 | 0.72 | 0.52 | 0.99 | 0.04 |
| TBX4 | 20.10 | <0.001 | 0.02 | 1.59 | 1.14 | 2.20 | 0.01 |
| TFPI | 38.82 | <0.001 | 1.52 | 2.07 | 1.45 | 2.94 | <0.001 |
| TGIF1 | 34.98 | <0.001 | 6.54 | 2.02 | 1.37 | 2.97 | <0.001 |
| TMEM71 | 42.34 | <0.001 | 0.44 | 2.16 | 1.54 | 3.02 | <0.001 |
| TMEM87B | 12.75 | <0.001 | 1.96 | 1.71 | 1.23 | 2.38 | <0.001 |
| TNK2 | 29.26 | <0.001 | 185.17 | 0.48 | 0.35 | 0.67 | <0.001 |
| TNNI3 | 13.43 | <0.001 | 1.45 | 0.69 | 0.51 | 0.95 | 0.02 |
| TP73 | 16.35 | <0.001 | 0.54 | 1.58 | 1.14 | 2.18 | 0.01 |
| TSPYL2 | 13.79 | <0.001 | 52.85 | 0.64 | 0.47 | 0.88 | 0.01 |
| TULP3 | 14.98 | <0.001 | 5.98 | 1.52 | 1.10 | 2.10 | 0.01 |
| WNT7B | 19.44 | <0.001 | 5.16 | 0.68 | 0.48 | 0.95 | 0.02 |
| ZCCHC4 | 13.74 | <0.001 | 2.33 | 1.72 | 1.24 | 2.39 | <0.001 |

Supplementary Table4. The intercept and coefficients of 71 genes of the optimal LASSO model

| Gene | Coefficient |
| --- | --- |
| (Intercept) |  |
| ALG6 | 0.014 |
| APEX2 | -0.006 |
| AQP7 | -0.098 |
| BCORL1 | 0.095 |
| BST1 | 0.124 |
| CA4 | -0.057 |
| CARHSP1 | 0.042 |
| CCT6A | 0.083 |
| CDH22 | 0.219 |
| CMTM1 | 0.024 |
| CNOT7 | 0.051 |
| COPA | 0.118 |
| COX16 | -0.013 |
| DDX25 | -0.017 |
| EIF3F | 0.114 |
| FASTKD5 | 0.016 |
| FMO5 | 0.058 |
| GBP5 | -0.105 |
| GDF10 | -0.136 |
| GIT1 | 0.044 |
| GLG1 | 0.216 |
| GRPR | 0.027 |
| HAUS1 | -0.041 |
| HIST1H3E | 0.136 |
| HIST2H4A | 0.106 |
| HLA.DQB1 | -0.094 |
| HSD17B7P2 | -0.124 |
| IL3RA | -0.076 |
| KCNK4 | -0.090 |
| KTI12 | -0.037 |
| LRRC16B | -0.238 |
| MBOAT4 | 0.035 |
| MEN1 | 0.219 |
| MRPL46 | -0.029 |
| MTIF2 | 0.054 |
| NAT6 | -0.015 |
| NUP107 | -0.001 |
| OAS1 | 0.013 |
| PACSIN2 | 0.077 |
| PALLD | -0.341 |
| PHACTR4 | -0.026 |
| PROX1 | 0.097 |
| PTCRA | 0.078 |
| PTPRN | 0.086 |
| RABGGTA | -0.053 |
| RAD18 | 0.142 |
| SAMD9 | 0.144 |
| SAP30 | 0.244 |
| SEC31A | 0.106 |
| SERF1A | -0.018 |
| SERTAD2 | 0.048 |
| SKI | 0.275 |
| SLC11A1 | 0.138 |
| SLC16A1 | 0.131 |
| SLC25A13 | -0.014 |
| SLC26A2 | 0.125 |
| SOX9 | 0.011 |
| SPATA6 | 0.065 |
| SULF2 | -0.046 |
| TBX4 | -0.218 |
| TFPI | 0.096 |
| TGIF1 | 0.035 |
| TMEM71 | 0.051 |
| TMEM87B | 0.036 |
| TNK2 | -0.015 |
| TNNI3 | 0.181 |
| TP73 | 0.020 |
| TSPYL2 | 0.016 |
| TULP3 | 0.032 |
| WNT7B | 0.106 |
| ZCCHC4 | 0.018 |

Supplementary Table5. Multivariate analyses between RFS and the 71-gene score in the TCGA and CGGA cohorts

| TCGA dataset | | | | CGGA dataset | | | |
| --- | --- | --- | --- | --- | --- | --- | --- |
| Clinical  feature | HR | 95%CI | P value | Clinical  feature | HR | 95%CI | P value |
| IDH1 | 0.36 | 0.24-0.53 | <0.001 | Histologic grade | 2.82 | 1.89-4.21 | <0.0001 |
| The 71-gene score | 2.08 | 1.39-3.09 | <0.001 | Chemotherapy | 1.11 | 0.75-1.65 | 0.59 |
|  |  |  |  | The 71-gene score | 2.48 | 1.75-3.52 | <0.0001 |

Notably, HR and CI refer to hazard ratio and confidence interval respectively.

Supplementary table6. Kaplan-Meier survival analysis results of the 71-gene score in subgroups of LGG patients stratified by clinical characteristics

| The TCGA dataset | | | | The CGGA dataset | | | |
| --- | --- | --- | --- | --- | --- | --- | --- |
| Variables | Group | Chisq value | P value | Variables | Group | Chisq value | P value |
| Age | >=41 | 30.86 | <0.001 | Age | >=40 | 22.55 | <0.001 |
|  | <41 | 2.34 | 0.13 |  | <40 | 7.78 | <0.01 |
| Tumour weight | >=248.5  <248.5 | 11.11  21.19 | <0.001  <0.001 |  |  |  |  |
| Gender | Female | 16.1 | <0.001 | Gender | Female | 3.16  25.44 | 0.08 |
|  | Male | 18.61 | <0.001 |  | Male |  | <0.001 |
| History of cancer | No | 4.97 | 0.03 | Codeletion_1p19q1 | No | 14.41  6.25 | <0.001 |
|  | Yes | 11.98 | <0.001 |  | Yes |  | 0.01 |
| Histologic grade | G2 | 7.74 | <0.01 | Histologic grade | G2 | 3.09  15.92 | 0.08 |
|  | G3 | 33.84 | <0.001 |  | G3 |  | <0.001 |
| *IDH1* mutation | Wild-type | 14.66 | <0.001 | IDH1 mutation | Wild-type | 10.17 | <0.01 |
|  | Mutant | 4.77 | 0.03 |  | Mutant | 20.13 | <0.001 |
| *TP53* mutation | Wild-type  Mutant | 24.45  7.5 | <0.001  <0.01 | MGMTp methylation | No  Yes | 8.52  28 | <0.01  <0.001 |
| Radiation therapy | No  Yes | 3.54  17.37 | 0.06  <0.001 | Radiation therapy | No  Yes | 13.3  16.57 | <0.001  <0.001 |
| Targeted therapy | No  Yes | 8.56  28.96 | <0.01  <0.001 | Chemotherapy | No  Yes | 7.64  20.31 | <0.001  <0.01 |

Supplementary table7. The significantly up-regulated signalling pathways in the low 71-gene score group of the CGGA cohort

| KEGG pathway name | Pathway size | Enrichement score | Normalized enrichment score | P value | Q value |
| --- | --- | --- | --- | --- | --- |
| KEGG_PATHWAYS_IN_CANCER | 307 | 0.42 | 1.69 | P<0.01 | 0.32 |
| KEGG_UBIQUITIN_MEDIATED_PROTEOLYSIS | 127 | 0.54 | 1.86 | P<0.01 | 0.42 |
| KEGG_RENAL_CELL_CARCINOMA | 65 | 0.53 | 1.80 | P<0.01 | 0.43 |
| KEGG_WNT_SIGNALING_PATHWAY | 143 | 0.44 | 1.67 | P<0.01 | 0.26 |
| KEGG_NON_SMALL_CELL_LUNG_CANCER | 53 | 0.48 | 1.71 | 0.01 | 0.38 |
| KEGG_TIGHT_JUNCTION | 116 | 0.38 | 1.56 | 0.01 | 0.19 |
| KEGG_TGF_BETA_SIGNALING_PATHWAY | 82 | 0.47 | 1.69 | 0.01 | 0.28 |
| KEGG_NEUROTROPHIN_SIGNALING_PATHWAY | 121 | 0.44 | 1.65 | 0.01 | 0.25 |
| KEGG_SMALL_CELL_LUNG_CANCER | 82 | 0.46 | 1.62 | 0.01 | 0.18 |
| KEGG_PANCREATIC_CANCER | 68 | 0.47 | 1.63 | 0.02 | 0.24 |
| KEGG_RIG_I_LIKE_RECEPTOR_SIGNALING_PATHWAY | 50 | 0.47 | 1.64 | 0.02 | 0.25 |
| KEGG_CHRONIC_MYELOID_LEUKEMIA | 71 | 0.51 | 1.72 | 0.02 | 0.44 |
| KEGG_ADHERENS_JUNCTION | 68 | 0.51 | 1.68 | 0.02 | 0.27 |
| KEGG_PROGESTERONE_MEDIATED_OOCYTE_MATURATION | 80 | 0.45 | 1.62 | 0.02 | 0.17 |
| KEGG_RNA_DEGRADATION | 49 | 0.55 | 1.72 | 0.02 | 0.58 |
| KEGG_SPHINGOLIPID_METABOLISM | 33 | 0.50 | 1.63 | 0.02 | 0.21 |
| KEGG_DORSO_VENTRAL_AXIS_FORMATION | 22 | 0.55 | 1.67 | 0.02 | 0.24 |
| KEGG_REGULATION_OF_ACTIN_CYTOSKELETON | 196 | 0.38 | 1.58 | 0.02 | 0.19 |
| KEGG_MAPK_SIGNALING_PATHWAY | 249 | 0.36 | 1.56 | 0.02 | 0.19 |
| KEGG_ENDOCYTOSIS | 171 | 0.39 | 1.60 | 0.02 | 0.18 |
| KEGG_INSULIN_SIGNALING_PATHWAY | 126 | 0.42 | 1.61 | 0.02 | 0.18 |
| KEGG_PURINE_METABOLISM | 144 | 0.35 | 1.49 | 0.03 | 0.18 |
| KEGG_NUCLEOTIDE_EXCISION_REPAIR | 44 | 0.54 | 1.70 | 0.03 | 0.33 |
| KEGG_COLORECTAL_CANCER | 61 | 0.50 | 1.62 | 0.03 | 0.19 |
| KEGG_PATHOGENIC_ESCHERICHIA_COLI_INFECTION | 51 | 0.50 | 1.63 | 0.03 | 0.22 |
| KEGG_PROSTATE_CANCER | 85 | 0.42 | 1.54 | 0.03 | 0.19 |
| KEGG_THYROID_CANCER | 29 | 0.50 | 1.63 | 0.03 | 0.19 |
| KEGG_GAP_JUNCTION | 82 | 0.42 | 1.57 | 0.03 | 0.19 |
| KEGG_ADIPOCYTOKINE_SIGNALING_PATHWAY | 64 | 0.41 | 1.55 | 0.03 | 0.19 |
| KEGG_AXON_GUIDANCE | 126 | 0.39 | 1.53 | 0.04 | 0.19 |
| KEGG_ERBB_SIGNALING_PATHWAY | 83 | 0.43 | 1.57 | 0.04 | 0.19 |
| KEGG_CYSTEINE_AND_METHIONINE_METABOLISM | 32 | 0.44 | 1.50 | 0.04 | 0.18 |

Supplementary Table8. The comparison of clinical characteristics among the three subgroups of LGG patients in the TCGA dataset

| Variables | Group | Cluster1 | Cluster2 | Cluster3 | P value | Statistical method |
| --- | --- | --- | --- | --- | --- | --- |
| Age |  | 40.66 | 39.01 | 41.38 | P>0.05 | Student t test |
| Tumour weight |  | 309.62 | 345.61 | 311.08 | P>0.05 | Student t test |
| The 71-gene score |  | -0.77 | 0.56 | -0.53 | P<0.05 except for the comparison between Cluster1 VS Cluster3 | Student t test |
| Gender | Female | 58 | 112 | 54 | P>0.05 except for the comparison between Cluster2 VS Cluster3 | Fisher’s exact test |
|  | Male | 75 | 164 | 43 |  |  |
| History of cancer | No | 53 | 110 | 47 | P>0.05 | Fisher’s exact test |
|  | Yes | 34 | 70 | 25 |  |  |
| Histological type | Astrocytoma | 7 | 150 | 35 | P>0.05 | Fisher’s exact test |
|  | Oligoastrocytoma  Oligodendroglioma | 32  94 | 68  58 | 29  33 |  |  |
| Histologic grade | G2 | 76 | 106 | 61 | P<0.05 except for the comparison between Cluster1 VS Cluster3 | Fisher’s exact test |
|  | G3 | 57 | 169 | 36 |  |  |
| *IDH1* mutation | Wild-type | 14 | 85 | 14 | P<0.05 except for the comparison between Cluster1 VS Cluster3 | Fisher’s exact test |
|  | Mutant | 119 | 191 | 83 |  |  |
| *TP53* mutation | Wild-type  Mutant | 116  17 | 99  177 | 43  54 | P<0.05 except for the comparison between Cluster2 VS Cluster3 | Fisher’s exact test |
| Radiation therapy | No  Yes | 45  22 | 36  87 | 37  31 | P<0.05 except for the comparison between Cluster1 VS Cluster3 | Fisher’s exact test |
| Targeted therapy | No  Yes | 57  64 | 92  146 | 40  37 | P>0.05 except for the comparison between Cluster2 VS Cluster3 | Fisher’s exact test |
|  |  |  |  |  |  |  |

Spplementary Table9. The comparison of clinical characteristics among the three subgroups of LGG patients in the CGGA dataset

| Variables | Group | Cluster1 | Cluster2 | Cluster3 | P value | Statistical method |
| --- | --- | --- | --- | --- | --- | --- |
| Age |  | 40.66 | 39.01 | 41.38 | P>0.05 | Student t test |
| The 71-gene score |  | -0.97 | 1.33 | -0.38 | P<0.05 for all cases | Student t test |
| Gender | Female | 75 | 54 | 56 | P>0.05 except for the comparison between Cluster1 VS Cluster2 | Fisher’s exact test |
|  | Male | 75 | 88 | 72 |  |  |
| Grade | II | 60 | 50 | 62 | P>0.05 except for the comparison between Cluster2 VS Cluster3 | Fisher’s exact test |
|  | III | 90 | 92 | 66 |  |  |
| *IDH1* mutation | Wild-type | 18 | 44 | 32 | P>0.05 except for the comparison between Cluster1 VS Cluster2 | Fisher’s exact test |
|  | Mutant | 97 | 98 | 93 |  |  |
| 1p19q_codeletion | Wild-type  Mutant | 83  67 | 115  26 | 59  32 | P<0.05 except for the comparison between Cluster1 VS Cluster3 | Fisher’s exact test |
| MGMTp methylation | No | 47 | 45 | 37 | P>0.05 | Fisher’s exact test |
|  | Yes | 91 | 57 | 52 |  |  |
| Radiation therapy | No | 43 | 27 | 29 | P>0.05 | Fisher’s exact test |
|  | Yes | 102 | 111 | 95 |  |  |
| Chemotherapy | No | 46 | 45 | 38 | P>0.05 | Fisher’s exact test |
|  | Yes | 100 | 95 | 86 |  |  |
